# A quantitative treatment assessment for optimizing combined virotherapy-chemotherapy regimens against HPV-induced cervical cancer

**DOI:** 10.1371/journal.pone.0342672

**Published:** 2026-09-21

**Authors:** Salil Ghosh, Amit Kumar Bag, Satyajit Mukherjee, Xianbing Cao, Amarnath Chatterjee, Priti Kumar Roy

**Affiliations:** 1 School of Mathematics and Statistics, Beijing Technology and Business University, Beijing, P. R. China; 2 Department of Mathematics, Centre for Mathematical Biology and Ecology, Jadavpur University, Kolkata, West Bengal, India; 3 Department of Mathematics, K. L. S. College, Magadh University, Nawada, Bodh Gaya, Bihar, India; University of Rochester School of Medicine and Dentistry, UNITED STATES OF AMERICA

## Abstract

Cervical cancer is strongly associated with persistent infection by high-risk human papillomavirus (HPV) and continues to pose a major global health burden in recent times. Oncolytic virotherapy has emerged as a promising targeted strategy that selectively eliminates cancer cells while simultaneously activating host immune responses. In this study, a deterministic mathematical model is developed to investigate the dynamics of HPV-induced cervical cancer under oncolytic virotherapy, both as a standalone intervention and in combination with chemotherapy. The model captures the interactions among HPV-infected epithelial cells, cancer cells, oncolytic virus–infected cancer cells, free viral particles, and virus-specific cytotoxic T-lymphocytes. Fundamental qualitative properties of the system are established, and threshold conditions governing oncolytic virus persistence are identified. To elucidate the relative influence of biological and therapeutic parameters, a comprehensive global sensitivity analysis is performed using the extended Fourier amplitude sensitivity test (eFAST) and partial rank correlation coefficients. This analysis reveals key mechanisms regulating cancer cell dynamics and highlights parameters that critically shape treatment outcomes. An optimal control framework is further employed to assess time-dependent therapeutic strategies, providing insight into effective scheduling of virotherapy and chemotherapy while balancing treatment cost. Furthermore, numerical simulation-based evidences justify the analytical findings obtained in the study and demonstrate the comparative advantages of the combined strategy.

## 1 Introduction

Cervical cancer is a major malignancy primarily driven by persistent infection with high-risk human papillomavirus (HPV) [1,2]. Following infection of cervical epithelial cells, HPV induces oncogenic transformation through sustained disruption of normal cellular regulatory processes, leading to abnormal cell proliferation and the development of precancerous lesions [3]. Persistent infection significantly increases the risk of progression to invasive cervical cancer [4]. Epidemiological evidence indicates that high-risk HPV genotypes, particularly HPV-16 and HPV-18, account for the majority of cervical cancer cases worldwide [5].

Despite substantial advances in screening strategies and prophylactic vaccination programs [6], HPV-associated cervical cancer continues to impose a considerable global public health burden, particularly in low and middle-income countries [7]. Treatment of cervical cancer, as with many malignancies, remains challenging due to pronounced biological heterogeneity and complex tumor–host interactions [8]. The primary therapeutic objective is to eliminate the maximum possible number of cancer cells while minimizing collateral damage to surrounding healthy tissues [9]. Conventional treatment modalities, including surgery, chemotherapy, and radiotherapy, as well as newer approaches such as angiogenesis inhibitors and immunotherapy, often fail to achieve complete cancer cell eradication without inducing significant toxicity to surrounding healthy tissues [10]. These limitations underscore the essence for alternative or complementary therapeutic approaches applicable to both early-stage and advanced cervical cancer, including adjuvant treatment settings [11].

Virotherapy employing selectively oncolytic viruses (OVs), either as a monotherapy or in combination with chemotherapy, has emerged as a promising targeted approach for the treatment of CIN (Cervical Intraepithelial Neoplasia) lesions and cervical cancer [12]. In oncolytic virotherapy, genetically engineered viruses are designed to preferentially infect, replicate within, and destroy cancer cells while sparing normal tissues [13]. These viruses exert their anticancer effects through two principal mechanisms. First, viral replication within cancer cells ultimately leads to cell lysis, releasing progeny virions that can subsequently infect neighboring malignant cells [14]. Second, cancer cell lysis results in the release of cancer-associated antigens, thereby activating host immune responses, including antibody production and cytotoxic T-cell activation, which further enhance cancer cell elimination [15]. Beyond direct oncolytic activity, the interaction between oncolytic viruses and the host immune system plays a critical role in shaping therapeutic outcomes, rendering treatment dynamics highly nonlinear and strongly time-dependent [16].

Several classes of oncolytic viruses, including adenoviruses and herpes simplex viruses, have been explored for cervical cancer therapy and other malignancies [17]. Encouraging preclinical and clinical outcomes have demonstrated their ability to selectively target cancer cells and enhance anticancer immunity [18]. Notably, regulatory approval of oncolytic viral therapies in multiple countries highlights their growing clinical relevance and therapeutic potential [19].

Although experimental and clinical investigations highlight the therapeutic potential of oncolytic virotherapy, the intricate interactions among HPV infection dynamics, cancer growth, viral replication, and immune responses remain insufficiently understood [20]. Various mathematical modeling-based studies have been developed lately on several infectious diseases and as well on cervical cancer [21–26] but these above-mentioned issues were not addressed rigorously in the existing studies [27]. At these points, our present study focuses specifically and captures the existing research gaps through mathematical modeling which offers a rigorous and systematic framework to integrate these multiscale processes, examine threshold phenomena, and assess the impact of potential treatment strategies to cure HPV-induced infection [28].

Motivated by these considerations, the present study develops a deterministic mathematical framework to investigate the within-host dynamics of HPV-induced cervical cancer under oncolytic virotherapy, both as a standalone treatment and in combination with chemotherapy. The proposed model is first employed to examine disease progression and to identify key parameters governing system behavior through analytical investigation of equilibrium states and their stability properties. Building on this foundation, an optimal control formulation is introduced to assess therapeutic intervention strategies, and Pontryagin’s Maximum Principle is applied to derive optimal treatment regimens. Numerical simulations are performed using MATLAB16 and Python to validate the analytical results and to explore treatment outcomes under different scenarios, and some crucial findings are discussed in relation to the results of existing studies.

## 2 Formulation of the mathematical model

**Fig 1:**
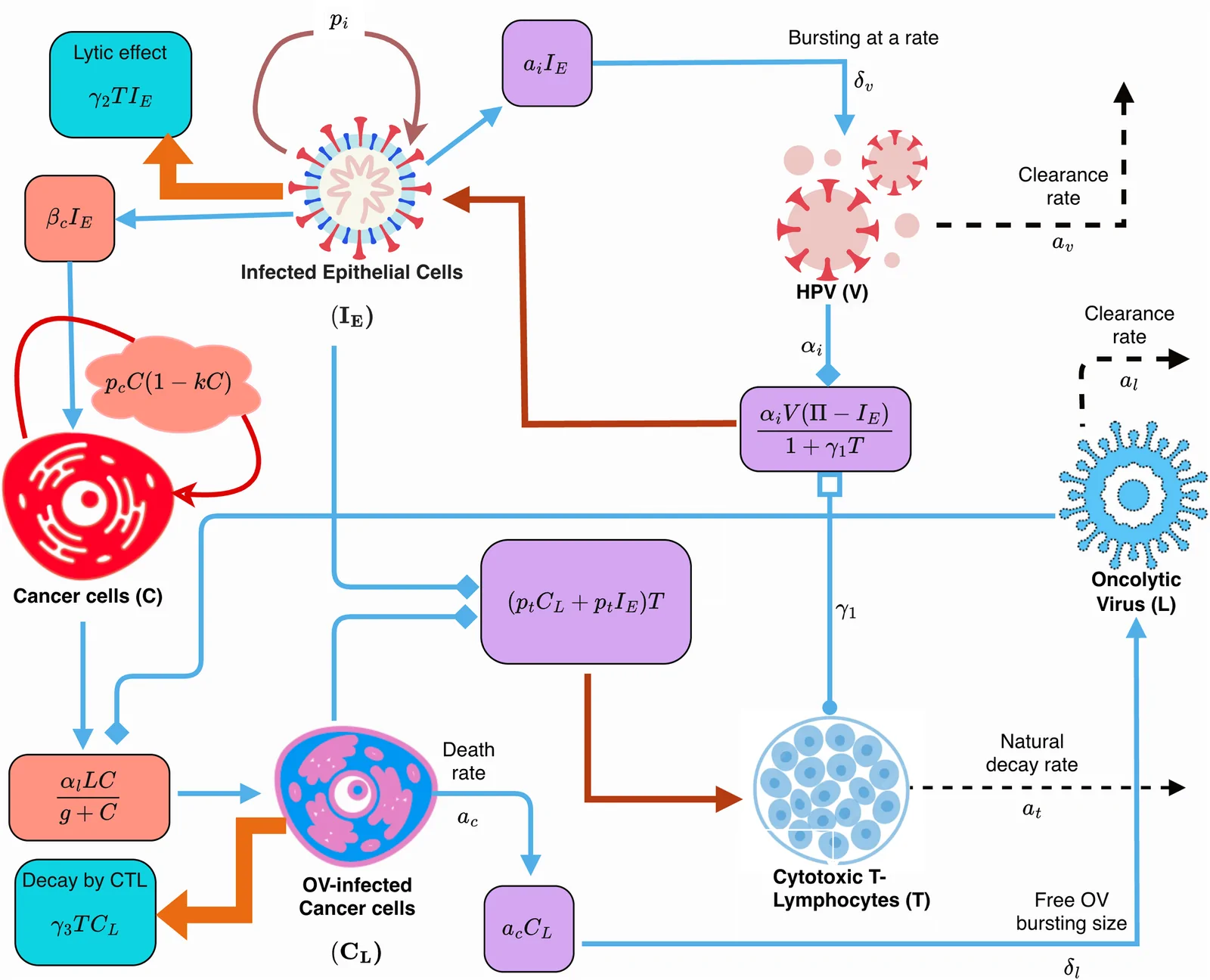
Schematic diagram demonstrating the interactions between the model state populations for system (1).

To formulate an ODE-based mathematical model of cervical cancer and its treatment using oncolytic virotherapy, we consider six state populations. We denote the HPV infected epithelium cells in the cervix as $I_{E}$, human papillomavirus (HPV) as *V*, cancer cells in the cervix as *C*, oncolytic virus-induced infected cancer cells as $C_{L}$, oncolytic viruses (OVs) as *L* and virus-specific cytotoxic T-lymphocytes (CTLs) as *T*. Nextly, we demonstrate the definitions of the system parameters and the interactions between the system variables which is further described in the schematic diagram represented in Fig 1.

$A_{1}$: Let Π represent the concentration of healthy epithelial cells in the cervix before infection by HPV. During interaction with HPV, healthy epithelial cells in the cervix become infected, denoted by $I_{E}$, at a rate of $\alpha_{i}$. Due to the non-lytic effect, CTLs inhibit HPV replication indicated by the term $\left(1+\gamma_{1}T\right)$. The concentration of uninfected cells in the cervix is represented by $\left(\Pi -I_{E}\right)$. This representation implicitly assumes that the total epithelial cell population remains constant over the time scale of infection, a simplifying assumption that is widely adopted in mathematical modeling of HPV-related cervical cancer dynamics. Infected cells proliferate at a rate of $p_{i}$, and die naturally at a rate of $a_{i}$. Infected cells transform into cancerous cells at a rate of $\beta_{c}$. For the lytic effect, CTL kills HPV-infected cells at a rate of $\gamma_{2}$.

$A_{2}$: Free HPV virions are produced from dead infected cells by bursting at a rate of $\delta_{v}$, and the clearance rate of free virions is indicated by $a_{v}$. New virions are not released from cancerous cells.
$A_{3}$: We assume that the cancer cells grow logistically at the rate $p_{c}$ with maximum burden (carrying capacity) $\frac{1}{k}$ due to limited nutrients, oxygen, and space. OVs infect cancer cells at a rate denoted by $\alpha_{l}$. However, due to their suppressive nature, cancer cells impede the function of OVs. This interaction is represented using the Michaelis-Menten kinetic form $-\frac{\alpha_{l}LC}{g+C}$, where *g* is the inhibitor coefficient.
$A_{4}$: $a_{c}$ is the death rate of infected cancer cells and CTLs kill infected cancerous cells at the rate $\gamma_{3}$.
$A_{5}$: Free OV particles released from dead infected cancer cells at the rate $\delta_{l}$ and free OV virions gets cleared at the rate $a_{l}$.
$A_{6}$: After encountering the HPV-infected cells and infected cancer cells, proliferation CTLs is denoted by the term $\left(p_{t}I_{E}+p_{t}C_{L}\right)$ and the CTLs naturally decay at a rate of $a_{t}$.

Thus, according to the assumptions made above, we have formulated the following six-dimensional mathematical model:


$$\begin{array}{cc} \frac{dI_{E}}{dt} & =\frac{\alpha_{i}V(\Pi -I_{E})}{1+\gamma_{1}T}+p_{i}I_{E}-a_{i}I_{E}-\beta_{c}I_{E}-\gamma_{2}TI_{E},\\ \frac{dV}{dt} & =\delta_{v}a_{i}I_{E}-a_{v}V,\\ \frac{dC}{dt} & =\beta_{c}I_{E}+p_{c}C(1-kC)-\frac{\alpha_{l}LC}{g+C},\\ \frac{dC_{L}}{dt} & =\frac{\alpha_{l}LC}{g+C}-a_{c}C_{L}-\gamma_{3}TC_{L},\\ \frac{dL}{dt} & =\delta_{l}a_{c}C_{L}-a_{l}L,\\ \frac{dT}{dt} & =(p_{t}I_{E}+p_{t}C_{L})T-a_{t}T. \end{array}$$ (1)

Here, we have considered the initial conditions as:


$$\begin{array}{cc} & I_{E}(0)=I_{E_{0}}>0,V(0)=V_{0}>0,C(0)=C_{0}\geq 0,C_{L}(0)=C_{L_{0}}\geq 0,L(0)=L_{0}\geq 0\\ & \text{and }T(0)=T_{0}\geq 0. \end{array}$$ (2)

## 3 Basic properties of the model

### 3.1 Non-negativity and boundedness

To establish that the proposed model represented by system (1) is mathematically consistent and biologically meaningful, we first demonstrate that all its solutions remain non-negative and bounded for any time t≥0 which is discussed in detail in the following two theorems.

**Theorem 1.** *Let* $\left(I_{E},V,C,C_{L},L,T\right)$ *be any solution of the system (1) along with the initial conditions denoted in (2). Then, the solution* $\left(I_{E},V,C,C_{L},L,T\right)$ *will be non-negative in* ${\mathbb{R}}_{+}^{6}$ *with* t≥0*.*

*Proof.* From the first equation of system (1), we have


$$\begin{array}{c} \frac{dI_{E}}{dt}=\frac{\alpha_{i}V(\Pi -I_{E})}{1+\gamma_{1}T}+p_{i}I_{E}-a_{i}I_{E}-\beta_{c}I_{E}-\gamma_{2}TI_{E}\geq p_{i}I_{E}-a_{i}I_{E}-\beta_{c}I_{E}-\gamma_{2}TI_{E}. \end{array}$$

Solving the above equation while using the result mentioned in [29], we get


$$\begin{array}{c} I_{E}(t)\geq I_{E_{0}}e^{\int_{0}^{t}\{p_{i}-a_{i}-\beta_{c}-\gamma_{2}T\}\;ds}>0. \end{array}$$

Similarly, from the remaining equations of system (1), we have that


$$\begin{array}{cc} V(t) & \geq V_{0}e^{-a_{v}t}>0,\\ C(t) & \geq C_{0}e^{\int_{0}^{t}\{p_{c}(1-kC)-\frac{\alpha_{l}L}{g+C}\}\;ds}\geq 0,\\ C_{L}(t) & \geq C_{L_{0}}e^{-\int_{0}^{t}\{a_{c}+\gamma_{3}T\}\;ds}\geq 0,\\ L(t) & \geq L_{0}e^{-a_{l}t}\geq 0\text{ and}\\ T(t) & \geq T_{0}e^{-a_{t}t}\geq 0. \end{array}$$

Therefore, the solution $\left(I_{E},V,C,C_{L},L,T\right)$ will be non-negative in ${\mathbb{R}}_{+}^{6}$ for all time t≥0. Hence, the proof is completed. □

**Theorem 2.** *Let* $\left(I_{E},V,C,C_{L},L,T\right)$ *be any solution of the system (1) with initial condition (2) in* ${\mathbb{R}}_{+}^{6}$. *Then, all the solutions are uniformly bounded for* t≥0*. Moreover, the set*


$$\begin{array}{c} 𝒟=\{(I_{E},V,C,C_{L},L,T)\in {\mathbb{R}}_{+}^{6}:I_{E},V,C,C_{L},L,T\leq \delta \text{ for some positive }\delta \} \end{array}$$

*is a positively invariant and absorbing set for system (1).*

*Proof.* To prove the theorem, we first assume that


$$\begin{array}{c} N(t)=\gamma_{3}p_{t}I_{E}(t)+V(t)+\gamma_{2}p_{t}C(t)+\gamma_{2}p_{t}C_{L}(t)+\gamma_{3}\gamma_{2}T(t)+L(t). \end{array}$$

Differentiating *N*(*t*) along solutions of (1), we obtain


$$\begin{array}{cc} \frac{dN}{dt} & \leq \alpha_{i}\gamma_{3}p_{t}\Pi \;V+\gamma_{3}p_{t}p_{i}I_{E}-\gamma_{3}p_{t}a_{i}I_{E}-\gamma_{3}p_{t}\beta_{c}I_{E}+\delta_{v}a_{i}I_{E}-a_{v}V\\ & \;+\gamma_{2}p_{t}\beta_{c}I_{E}+\gamma_{2}p_{t}p_{c}C-\gamma_{2}p_{t}p_{c}kC^{2}-\gamma_{2}p_{t}a_{c}C_{L}-\gamma_{3}\gamma_{2}a_{t}T+\delta_{l}a_{c}C_{L}-a_{l}L\\ & \leq -nN-\gamma_{2}p_{t}p_{c}k(C-\frac{1}{k}{)}^{2}+\frac{\gamma_{2}p_{t}p_{c}}{k}, \end{array}$$ (3)

where


$$\begin{array}{c} n=\min\{(a_{v}-\alpha_{i}\gamma_{3}p_{t}\Pi ),(a_{i}-p_{i}-\frac{\delta_{v}a_{i}}{\gamma_{3}p_{t}}-\frac{\gamma_{2}\beta_{c}}{\gamma_{3}}),a_{l},p_{c},a_{t},(a_{c}-\frac{\delta_{l}a_{c}}{\gamma_{2}p_{t}})\}>0. \end{array}$$

Dropping the non-positive quadratic term in (3) yields


$$\begin{array}{c} \frac{dN}{dt}+nN\leq \frac{\gamma_{2}p_{t}p_{c}}{k}. \end{array}$$

Multiplying by $e^{nt}$ and integrating from 0 to *t*, we obtain


$$\begin{array}{c} N(t)\leq N(0)e^{-nt}+\frac{\gamma_{2}p_{t}p_{c}}{nk}(1-e^{-nt}), \end{array}$$

and hence,


$$\begin{array}{c} N(t)\leq \max\{N(0),\frac{\gamma_{2}p_{t}p_{c}}{nk}\}\;\text{for all }t\geq 0, \end{array}$$

which implies ${lim sup}_{t\to \infty }N(t)\leq \frac{\gamma_{2}p_{t}p_{c}}{nk}$.

Finally, since all the state variables are non-negative, each component is bounded above by *N*(*t*), namely


$$\begin{array}{c} I_{E}(t)\leq \frac{N(t)}{\gamma_{3}p_{t}},\;V(t)\leq N(t),\;C(t)\leq \frac{N(t)}{\gamma_{2}p_{t}},\;C_{L}(t)\leq \frac{N(t)}{\gamma_{2}p_{t}},\;T(t)\leq \frac{N(t)}{\gamma_{3}\gamma_{2}},\;L(t)\leq N(t). \end{array}$$

Let,


$$\begin{array}{c} \delta =max\{\frac{\gamma_{2}p_{c}}{\gamma_{3}nk},\frac{\gamma_{2}p_{t}p_{c}}{nk},\frac{p_{c}}{nk},\frac{p_{t}p_{c}}{\gamma_{3}nk}\} \end{array}$$

Therefore, all solutions initiating in ${\mathbb{R}}_{+}^{6}$ enter and remain in the bounded region


$$\begin{array}{c} 𝒟=\{(I_{E},V,C,C_{L},L,T)\in {\mathbb{R}}_{+}^{6}:I_{E},V,C,C_{L},L,T\leq \delta \}, \end{array}$$

and hence 𝒟 is bounded. Thus, 𝒟 is a positively invariant and absorbing set for the system (1), and all solutions initiating in ${\mathbb{R}}_{+}^{6}$ remain uniformly bounded. □

### 3.2 Oncolytic virus-free equilibrium (OFE) and basic reproduction number ${ℛ}_{0}$

The oncolytic virus-free equilibrium point (OFEP) *E*_0_ of system (1) is denoted as *E*_0_ and it is defined as: $E_{0}=(I_{E}^{\prime },V^{\prime },C^{\prime },0,0,T^{\prime })$ where


$$\begin{array}{cc} & I_{E}^{\prime }=\frac{a_{t}}{p_{t}},\;V^{\prime }=\frac{\delta_{v}a_{i}a_{t}}{a_{v}p_{t}},\;C^{\prime }=\frac{1}{2k}\left(1+\sqrt{1+\frac{4k\beta_{c}a_{t}}{p_{c}p_{t}}}\right)\text{ and }T^{\prime }=\frac{1}{2b_{1}}\left(-b_{2}+\sqrt{(b_{2}{)}^{2}-4b_{1}b_{3}}\right).\\ & \text{Here, }b_{1}=\gamma_{1}\gamma_{2}a_{v},\;b_{2}=a_{v}(\gamma_{2}+\gamma_{1}\beta_{c}+\gamma_{1}a_{i}-\gamma_{1}p_{i}),\\ & b_{3}=a_{v}\beta_{c}+a_{v}a_{i}-a_{v}p_{i}-\alpha_{i}\delta_{v}a_{i}\left(\Pi -\frac{a_{t}}{p_{t}}\right)\text{ and }\sqrt{(b_{2}{)}^{2}-4b_{1}b_{3}}>b_{2}. \end{array}$$ (a1)

In the context of cancer virotherapy, the basic reproduction number ${ℛ}_{0}$ of system (1) describes how effectively the oncolytic virus spreads within the cancer cells. If ${ℛ}_{0}>1$, the viral infection continues spreading, potentially helping to destroy more cancer cells. If ${ℛ}_{0}<1$, the infection fades, reducing its therapeutic impact. This threshold also helps us to determine the key parameters influencing cancer cell infection dynamics. We determine the ${ℛ}_{0}$ of system (1) by applying the next generation matrix approach [30,31]. Let, ℱ be the matrix of the terms leading to the infection by oncolytic viruses and 𝒱 be the matrix of other transfer terms in system (1) such that


$$\begin{array}{c} ℱ=(\begin{array}{c} \frac{\alpha_{l}LC}{g+C}\\ 0 \end{array})\text{ and }𝒱=(\begin{array}{c} a_{c}C_{L}+\gamma_{3}TC_{L}\\ a_{l}L-\delta_{l}a_{c}C_{L} \end{array}). \end{array}$$

Evaluating the Jacobian matrices of ℱ and 𝒱 at *E*_0_ produces *F* and *V* as described below:


$$\begin{array}{c} F=(\begin{array}{cc} 0 & \frac{\alpha_{l}C^{\prime }}{g+C^{\prime }}\\ 0 & 0 \end{array})\;\text{and}\;V=(\begin{array}{cc} a_{c}+\gamma_{3}T^{\prime } & 0\\ -\delta_{l}a_{c} & a_{l} \end{array}). \end{array}$$

Therefore,


$$\begin{array}{c} FV^{-1}=(\begin{array}{cc} \frac{\delta_{l}\alpha_{l}a_{c}C^{\prime }}{a_{l}\left(a_{c}+\gamma_{3}T^{\prime }\right)\left(g+C^{\prime }\right)} & \frac{\alpha_{l}C^{\prime }}{a_{l}\left(g+C^{\prime }\right)}\\ 0 & 0 \end{array}) \end{array}$$

and thus, the basic reproduction number for system (1) which is actually the largest eigenvalue (in modulus) of *FV*^−1^, can be written as


$$\begin{array}{c} {ℛ}_{0}=\frac{\delta_{l}\alpha_{l}a_{c}C^{\prime }}{a_{l}\left(a_{c}+\gamma_{3}T^{\prime }\right)\left(g+C^{\prime }\right)}. \end{array}$$ (4)

**Remark 1.** *In the present framework, the basic reproduction number* ${ℛ}_{0}$ *represents the average number of newly infected cancer cells generated by a single oncolytic virus-infected cancer cell during its lifetime. The numerator of* ${ℛ}_{0}$ *reflects the effective production and infection potential of oncolytic viruses through viral bursting and infection of susceptible cancer cells, while the denominator incorporates viral clearance and immune response mediated by cytotoxic T-lymphocytes. Thus*, ${ℛ}_{0}$ *serves as a key indicator of the ability of the oncolytic virus to establish and persist within the cancer microenvironment.*

**Remark 2.** *The value of* ${ℛ}_{0}$ *acts as a threshold parameter governing the qualitative dynamics of the system near the oncolytic virus-free equilibrium. When* ${ℛ}_{0}<1$, *the oncolytic virus population fails to invade the tumor and the oncolytic virus-free equilibrium is expected to be stable. In contrast, when* ${ℛ}_{0}>1$*, viral invasion becomes imminent, leading to sustained viral activity within cancer cells and potential destabilization of the oncolytic virus-free state.* In view of the threshold behavior induced by ${ℛ}_{0}$, we now proceed to investigate the local asymptotic stability of the oncolytic virus-free equilibrium.

**Theorem 3.** *Local asymptotic stability near the OFE* $E_{0}=(I_{E}^{\prime },V^{\prime },C^{\prime },0,0,T^{\prime })$ *for system (1) holds if and only if the following conditions are satisfied:*

1. ${ℛ}_{0}<1$,
2. ${𝒜}_{1}>0$, ${𝒜}_{3}>0$, ${𝒜}_{1}{𝒜}_{2}-{𝒜}_{3}>0$

*where*


$$\begin{array}{c} \left\{ \begin{array}{cc} {𝒜}_{1} & =a_{v}-a_{11}+a_{t}-p_{t}I_{E}^{\prime },\\ {𝒜}_{2} & =(a_{t}-p_{t}I_{E}^{\prime })(a_{v}-a_{11})+\frac{\delta_{v}a_{i}\alpha_{j}\left(\pi -I_{E}^{\prime }\right)\left(p_{t}I_{E}^{\prime }-a_{t}\right)}{1+\gamma_{1}T^{\prime }}-a_{11}a_{v}-p_{t}a_{16}T^{\prime },\\ {𝒜}_{3} & =\frac{\delta_{v}a_{i}\alpha_{j}\left(\pi -I_{E}^{\prime }\right)\left(p_{t}I_{E}^{\prime }-a_{t}\right)}{1+\gamma_{1}T^{\prime }}+\left(p_{t}I_{E}^{\prime }-a_{t}\right)a_{11}a_{v}-a_{v}p_{t}a_{16}T^{\prime }. \end{array}\right. \end{array}$$ (5)

*If any of these conditions is not satisfied, the system is considered to be unstable.*

*Proof.* The Jacobian matrix of system (1) at the OFEP *E*_0_ is given as


$$\begin{array}{c} J_{0}=(\begin{array}{cccccc} a_{11} & \frac{\alpha_{i}(\Pi -I_{E}^{\prime })}{1+\gamma_{1}T^{\prime }} & 0 & 0 & 0 & a_{16}\\ \delta_{v}a_{i} & -a_{v} & 0 & 0 & 0 & 0\\ \beta_{c} & 0 & a_{33} & 0 & \frac{-\alpha_{l}C^{\prime }}{g+C^{\prime }} & 0\\ 0 & 0 & 0 & \left(-a_{c}-\gamma_{3}T^{\prime }\right) & \frac{\alpha_{l}C^{\prime }}{g+C^{\prime }} & 0\\ 0 & 0 & 0 & \delta_{l}a_{c} & -a_{l} & 0\\ p_{t}T^{\prime } & 0 & 0 & p_{t}T^{\prime } & 0 & p_{t}I_{E}^{\prime }-a_{t} \end{array}) \end{array}$$

where $a_{11}=\frac{-\alpha_{i}V^{\prime }}{1+\gamma_{1}T^{\prime }}+p_{i}-a_{i}-\beta_{c}-\gamma_{2}T^{\prime }$, $a_{16}=\frac{-\gamma \alpha_{i}V^{\prime }\left(\Pi -I_{E}^{\prime }\right)}{{\left(1+\gamma_{1}T^{\prime }\right)}^{2}}-\gamma_{2}I_{E}^{\prime }$ and $a_{33}=p_{c}\left(1-2kC^{\prime }\right)$.

The characteristic equation of *J*_0_ is $|J_{0}-\lambda I_{6}|=0$. i.e., $(\lambda -a_{33})[\lambda^{2}+(a_{c}+a_{l}+\gamma_{3}T^{\prime })\lambda +a_{l}(a_{c}+\gamma_{3}T^{\prime })(1-{ℛ}_{0})][\lambda^{3}+{𝒜}_{1}\lambda^{2}+{𝒜}_{2}\lambda +{𝒜}_{3}=0]=0$

Now, solving the above equation, we get one eigenvalue of *J*_0_ as $\lambda =a_{33}=p_{c}(1-2kC^{\prime })=-p_{c}\sqrt{1+\frac{4k\beta_{c}a_{t}}{p_{c}p_{t}}}<0$, where the last inequality simply follows from the positivity of the model parameters. The other eigenvalues are roots of the following two equations:


$$\begin{array}{c} \lambda^{2}+(a_{c}+a_{l}+\gamma_{3}T^{\prime })\lambda +a_{l}(a_{c}+\gamma_{3}T^{\prime })(1-{ℛ}_{0})=0, \end{array}$$ (6)

$$\begin{array}{c} \text{and}\;\lambda^{3}+{𝒜}_{1}\lambda^{2}+{𝒜}_{2}\lambda +{𝒜}_{3}=0 \end{array}$$ (7)

where ${𝒜}_{1}$, ${𝒜}_{2}$ and ${𝒜}_{3}$ are indicated in (5). The quadratic equation (6) characterizes the local dynamics of the oncolytic virus-infected cancer cell and free virus subsystems around the oncolytic virus–free equilibrium.

Now, all roots of (6) have negative real parts if $a_{l}\left(a_{c}+\gamma_{3}T^{\prime }\right)\left(1-{ℛ}_{0}\right)$ is positive, i.e., if ${ℛ}_{0}<1$. Also, by Routh-Hurwitz criteria, all roots of equation (7) have negative real parts if ${𝒜}_{1}>0,$ ${𝒜}_{3}>0,$ ${𝒜}_{1}{𝒜}_{2}-{𝒜}_{3}>0$.

By the combination of these two set of conditions discussed above, we can say that when ${ℛ}_{0}<1$ and the Routh–Hurwitz conditions ${𝒜}_{1}>0$, ${𝒜}_{3}>0$, and ${𝒜}_{1}{𝒜}_{2}-{𝒜}_{3}>0$ are satisfied, all eigenvalues of the Jacobian matrix *J*_0_ have negative real parts. Therefore, the oncolytic virus-free equilibrium *E*_0_ is locally asymptotically stable. □

### 3.3 Endemic equilibrium state and stability analysis

In this section, we derive the endemic equilibrium state of system (1), which corresponds to the persistent presence of infection within the host. We then analyze the local stability of this equilibrium by examining the associated Jacobian matrix.

#### 3.3.1 Endemic equilibrium state.

The endemic equilibrium point (EEP) of system (1) is denoted as $E_{n}$ and it represents a biologically meaningful steady state in which HPV infection, cancer cells, and oncolytic viruses coexist within the host. The existence of such an equilibrium is typically associated with parameter regimes where viral persistence is possible, complementing the threshold behavior characterized by ${ℛ}_{0}$ in the previous section.

We have $E_{n}=(I_{E}^{*},V^{*},C^{*},C_{L}^{*},L^{*},T^{*})$ where


$$\begin{array}{cc} I_{E}^{*}=\; & \pi +\frac{\left[\gamma_{2}a_{c}(1-\alpha_{l}\delta_{l}a_{c})C^{*}+g+\gamma_{3}a_{l}(g+C^{*})(p_{i}-a_{i}-\beta_{c})\right]}{\alpha_{i}\delta_{v}a_{i}\gamma_{3}^{2}a_{l}^{2}(g+C^{*}{)}^{2}}[a_{v}(a_{l}\gamma_{3}+\\ & \gamma_{1}a_{c}^{2}\alpha_{l}\delta_{l}-1)C^{*}+a_{v}g(a_{l}\gamma_{3}-a_{c}\gamma_{1})],\\ V^{*}=\; & \frac{\delta_{v}a_{i}}{a_{v}}I_{E}^{*};\;C_{L}^{*}=\frac{a_{t}}{p_{t}}-I_{E}^{*};\;L^{*}=\frac{\delta_{l}a_{c}}{a_{l}}\left(\frac{a_{t}}{p_{t}}-I_{E}^{*}\right);\;T^{*}=\frac{a_{c}}{\gamma_{3}}\left[\frac{\alpha_{l}\delta_{l}C^{*}}{a_{l}(g+C^{*})}-1\right]. \end{array}$$

Here, $C^{*}$ is a positive root of the equation $\psi_{0}C^{5}+\psi_{1}C^{4}+\psi_{2}C^{3}+\psi_{3}C^{2}+\psi_{4}C+\psi_{5}=0$ and the coefficients of this equation are given as


$$\begin{array}{cc} \psi_{0} & =\alpha_{i}\delta_{v}a_{i}p_{c}k\gamma_{3}^{2}a_{l}^{3},\;\psi_{1}=\alpha_{i}\delta_{v}a_{i}p_{c}\gamma_{3}^{2}a_{l}^{3}(3kg-1),\\ \psi_{2} & =\alpha_{i}\delta_{v}a_{i}\gamma_{3}^{2}a_{l}^{3}\left(3p_{c}ka_{l}g^{2}-3p_{c}a_{l}g+\frac{\alpha_{l}\delta_{l}a_{c}a_{t}}{p_{t}}-\alpha_{l}\delta_{l}a_{c}\Pi -a_{l}\beta_{c}\Pi \right)\\ & +(\alpha_{l}\delta_{l}a_{c}+a_{l}\beta_{c})b_{1},\\ \psi_{3} & =\alpha_{i}\delta_{v}a_{i}\gamma_{3}^{2}a_{l}^{3}\left(p_{c}ka_{l}g^{3}+\frac{2\alpha_{l}\delta_{l}a_{c}a_{t}g}{p_{t}}-3p_{c}a_{l}g^{2}\right)+2\left(\alpha_{l}\delta_{l}a_{c}+a_{l}\beta_{c}\right)g\Pi \\ & -\left(\alpha_{l}\delta_{l}a_{c}+a_{l}\beta_{c}\right)b_{2}+a_{l}g\beta_{c}b_{1},\\ \psi_{4} & =\alpha_{i}\delta_{v}a_{i}\gamma_{3}^{2}a_{l}^{2}g^{2}\left(\frac{\alpha_{l}\delta_{l}a_{c}a_{t}}{p_{t}}-p_{c}a_{l}g-\alpha_{l}\delta_{l}a_{c}\Pi -a_{l}\beta_{c}\Pi \right)-\left(\alpha_{l}\delta_{l}a_{c}+a_{l}\beta_{c}\right)b_{3}+a_{l}\beta_{c}gb_{2},\\ \psi_{5} & =a_{l}\beta_{c}gb_{3},\\ b_{1} & =\left[\gamma_{2}a_{c}(\alpha_{l}\delta_{l}a_{c}-1)+\gamma_{3}a_{l}(a_{i}+\beta_{c}-p_{i})\right]a_{v}\left(a_{l}\gamma_{3}+\gamma_{1}a_{c}^{2}\alpha_{l}\delta_{l}-1\right),\\ b_{2} & =(a_{l}\gamma_{3}g-\gamma_{1}a_{c}g)(\gamma_{2}\alpha_{l}\delta_{l}a_{c}^{2}-\gamma_{2}a_{c}+\gamma_{3}a_{l}a_{i}+\gamma_{3}a_{l}\beta_{c}-\gamma_{3}a_{l}p_{i})\\ & +g(a_{l}a_{v}\gamma_{3}+\gamma_{1}a_{v}a_{c}^{2}\alpha_{l}\delta_{l}-a_{v})(\gamma_{3}a_{l}a_{i}+\gamma_{3}a_{l}\beta_{c}-\gamma_{3}a_{l}p_{i}-1)\text{ and }\\ b_{3} & =g^{2}(\gamma_{1}a_{c}-\gamma_{3}a_{l})(\gamma_{3}a_{l}a_{i}+\gamma_{3}a_{l}\beta_{c}-\gamma_{3}a_{l}p_{i}-1). \end{array}$$

To address the existence and uniqueness of the biologically meaningful endemic equilibrium, we now analyze the quintic polynomial governing the steady-state cancer cell population $C^{*}$. In particular, we establish sufficient conditions under which this polynomial admits a unique positive root, thereby ensuring the uniqueness of the endemic equilibrium.

**Theorem 4.** *Let*


$$\begin{array}{c} f(C)=\psi_{0}C^{5}+\psi_{1}C^{4}+\psi_{2}C^{3}+\psi_{3}C^{2}+\psi_{4}C+\psi_{5},\;C>0, \end{array}$$

*where the coefficients* $\psi_{i}$ *are defined earlier. It can be verified that, under the baseline parameter set provided in* Table 1*, the coefficients satisfy*


$$\begin{array}{cc} & \psi_{0}>0, \end{array}$$ (8)

$$\begin{array}{cc} & \psi_{1}>0,\;\psi_{2}>0,\;\psi_{3}>0,\;\psi_{4}>0, \end{array}$$ (9)

$$\begin{array}{cc} & \psi_{5}<0. \end{array}$$ (10)

**Table 1:** Model parameters, biological interpretations, baseline values, and corresponding sources used in the numerical simulations of the cervical cancer–oncolytic virotherapy model.

Parameter	Definition	Ranges/values (unit)	Source
Π	Concentration of epithelial cells	10^4^ cells mm^-3^	Assumed
$\alpha_{i}$	Infection rate of epithelial cells	0.0067 mm^3^ virions^-1^ day^-1^	[32,33]
$p_{i}$	Proliferation rate of infected epithelial cells	0.01 day^-1^	[21,34]
$a_{i}$	Death rate of infected cells	0.0001 day^-1^	[32]
$\beta_{c}$	Rate at which infected cells gets transformed into cancer cells	0.008 day^-1^	[35]
$\gamma_{1}$	Rate of non-lytic effect	0.01 mm^3^ cells^-1^	[36]
$\gamma_{2}$	Rate of lytic effect	0.01 mm^3^ cells^-1^ day^-1^	[36]
$\delta_{v}$	Free HPV virions bursting size	1000 virions cells^-1^	[33]
$a_{v}$	Free HPV virions clearance rate	0.03 day^-1^	[33,37]
$p_{c}$	Cancerous cells proliferation rate	0.2 day^-1^	[37]
*k*	Inverse of maximum burden of cancer cells proliferation	10^−7^ mm^3^ cells^-1^	Assumed
$\alpha_{l}$	OV-induced infection rate of cancerous cells	0.001 cells mm^-3^ virions^-1^ day^-1^	[38]
*g*	Inhibitor coefficient	10^7^ cells mm^-3^	[1]
$a_{c}$	Natural death rate of cancer cells	0.5 day^-1^	[21]
$\gamma_{3}$	Decay rate infected cancerous cells by effector cells	0.1 mm^3^ cells^-1^ day^-1^	[39]
$\delta_{l}$	Free OV virions bursting size	10^3^ virions cells^-1^	[38]
$a_{l}$	Free OV virions clearance rate	0.01 day^-1^	[38]
$p_{t}$	Proliferation rate of T	0.01 mm^3^ cells^-1^ day^-1^	[39]
$a_{t}$	Decay rate of T	0.01 day^-1^	[39]
$d_{i}$	Decay rate of infected cells due to chemotherapy	0.06 day^-1^	Assumed
$d_{c}$	Decay rate of cancer cells due to chemotherapy	0.06 day^-1^	[21]
$d_{l}$	Decay rate of infected cancer cells due to chemotherapy	0.06 day^-1^	[40]
$d_{t}$	Decay rate of T due to chemotherapy	0.06 day^-1^	Assumed
$\gamma_{4}$	Rate at which concentration of chemotherapeutic drug decay	0.9 day^-1^	[40]

*Then the equation f(C) = 0 admits a unique positive root* $C^{*}>0$.

*Consequently, the endemic equilibrium* $E_{n}=(I_{E}^{*},V^{*},C^{*},C_{L}^{*},L^{*},T^{*})$ *exists uniquely in the biologically feasible region, provided in addition that*


$$\begin{array}{c} 0<I_{E}^{*}<\frac{a_{t}}{p_{t}}\;\text{and}\;\frac{\alpha_{l}\delta_{l}C^{*}}{a_{l}(g+C^{*})}>1. \end{array}$$

*Proof.* Since all the model parameters are positive, it follows that


$$\begin{array}{c} \psi_{0}=\alpha_{i}\delta_{v}a_{i}p_{c}k\gamma_{3}^{2}a_{l}^{3}>0. \end{array}$$

Hence,


$$\begin{array}{c} \lim_{C\to +\infty }f(C)=+\infty . \end{array}$$

Also, by (10),


$$\begin{array}{c} f(0)=\psi_{5}<0. \end{array}$$

Therefore, by continuity of *f*(*C*), there exists at least one $C^{*}>0$ such that $f(C^{*})=0$.

Now differentiating *f*(*C*) with respect to *C*, we obtain that


$$\begin{array}{c} f^{\prime }(C)=5\psi_{0}C^{4}+4\psi_{1}C^{3}+3\psi_{2}C^{2}+2\psi_{3}C+\psi_{4}. \end{array}$$

Using (8) – (9), we obtain


$$\begin{array}{c} f^{\prime }(C)>0\;\text{for all }C>0. \end{array}$$

Thus, *f* is strictly increasing on (0,∞). Hence *f*(*C*) can cross the horizontal axis at most once on (0,∞).

Combining existence with strict monotonicity, we conclude that *f*(*C*) = 0 has exactly one positive root $C^{*}>0$.

Once $C^{*}$ is uniquely determined, the remaining equilibrium components


$$\begin{array}{c} V^{*}=\frac{\delta_{v}a_{i}}{a_{v}}I_{E}^{*},\;C_{L}^{*}=\frac{a_{t}}{p_{t}}-I_{E}^{*},\;L^{*}=\frac{\delta_{l}a_{c}}{a_{l}}\left(\frac{a_{t}}{p_{t}}-I_{E}^{*}\right),\;T^{*}=\frac{a_{c}}{\gamma_{3}}\left(\frac{\alpha_{l}\delta_{l}C^{*}}{a_{l}(g+C^{*})}-1\right) \end{array}$$

are uniquely determined.

Moreover, under the adopted baseline parameter set, the conditions


$$\begin{array}{c} 0<I_{E}^{*}<\frac{a_{t}}{p_{t}}\;\text{and}\;\frac{\alpha_{l}\delta_{l}C^{*}}{a_{l}(g+C^{*})}>1 \end{array}$$

are satisfied, ensuring that $C_{L}^{*}>0$, $L^{*}>0$, and $T^{*}>0$.

Therefore, the endemic equilibrium is unique and biologically feasible. □

**Remark 3.** *The above result provides a sufficient condition for the existence and uniqueness of the biologically meaningful endemic equilibrium. In particular, under the baseline parameter set used in the numerical simulations, the quintic polynomial governing* $C^{*}$ *is strictly increasing on* (0,∞) *and admits a unique positive root. This ensures that the equilibrium analyzed throughout this study is uniquely defined.*

#### 3.3.2 Stability analysis.

In this subsection, we investigate the local asymptotic stability of the endemic equilibrium point (EEP) $E_{n}=(I_{E}^{*},V^{*},C^{*},C_{L}^{*},L^{*},T^{*})$ of system (1). The analysis is carried out by linearizing the system around $E_{n}$ and examining the eigenvalues of the associated Jacobian matrix. The Routh-Hurwitz stability criteria are then employed to derive sufficient conditions under which all eigenvalues have negative real parts.

The Jacobian of system (1) at EEP, $E_{n}$ is given by


$$\begin{array}{c} J_{n}=(\begin{array}{cccccc} a_{11} & \frac{\alpha_{i}(\Pi -I_{E}^{*})}{1+\gamma_{1}T^{*}} & 0 & 0 & 0 & a_{16}\\ \delta_{v}a_{i} & -a_{v} & 0 & 0 & 0 & 0\\ \beta_{c} & 0 & a_{33} & 0 & -\frac{\alpha_{l}C^{*}}{g+C^{*}} & 0\\ 0 & 0 & \frac{\alpha_{l}gL^{*}}{(g+C^{*}{)}^{2}} & \left(-a_{c}-\gamma_{3}T^{*}\right) & \frac{\alpha_{l}C^{*}}{g+C^{*}} & -\gamma_{3}C_{L}^{*}\\ 0 & 0 & 0 & \delta_{l}a_{c} & -a_{l} & 0\\ p_{t}T^{*} & 0 & 0 & p_{t}T^{*} & 0 & p_{t}(I_{E}^{*}+C_{L}^{*})-a_{t} \end{array}) \end{array}$$

where $a_{11}=-\frac{\alpha_{i}V^{*}}{1+\gamma_{1}T^{*}}+p_{i}-a_{i}-\beta_{c}-\gamma_{2}T^{*}$, $a_{16}=-\frac{\gamma_{1}\alpha_{i}V^{*}(\Pi -I_{E}^{*})}{(1+\gamma_{1}T^{*}{)}^{2}}-\gamma_{2}I_{E}^{*}$ and $a_{33}=p_{c}(1-2kC^{*})-\frac{\alpha_{l}gL^{*}}{(g+C^{*}{)}^{2}}$.

The characteristic equation associated with $J_{n}$ is given by


$$\begin{array}{c} \lambda^{6}+{ℬ}_{1}\lambda^{5}+{ℬ}_{2}\lambda^{4}+{ℬ}_{3}\lambda^{3}+{ℬ}_{4}\lambda^{2}+{ℬ}_{5}\lambda +{ℬ}_{6}=0 \end{array}$$ (11)

where


$$\begin{array}{cc} {ℬ}_{1} & =a_{v}+a_{l}+a_{c}+a_{t}+\gamma_{3}T^{*}-a_{11}-p_{t}I_{E}^{*}-p_{t}C_{L}^{*}-a_{33},\\ {ℬ}_{2} & =-\frac{\delta_{v}a_{i}\alpha_{i}(\Pi -I_{E}^{*})}{\left(1+\gamma_{1}T^{*}\right)}+(a_{v}-a_{11})(a_{l}+a_{c}+a_{t}+\gamma_{3}T^{*}-p_{t}I_{E}^{*}-p_{t}C_{L}^{*}-a_{33})\\ & +a_{l}(a_{c}+\gamma_{3}T^{*})-\delta_{l}a_{c}\alpha_{l}\frac{C^{*}}{g+C^{*}}+a_{t}(a_{l}+a_{c}+\gamma_{3}T^{*})-p_{t}(I^{*}+C_{L}^{*})(a_{l}+a_{c}+\gamma_{3}T^{*})\\ & +\delta_{v}\delta_{l}\frac{a_{i}\alpha_{i}\alpha_{l}^{2}a_{c}gC^{*}L^{*}(\pi -I_{E}^{*})}{(1+\gamma_{1}T^{*})(g+C^{*}{)}^{2}}+p_{t}\gamma_{3}T^{*}C_{L}^{*}-a_{33}(a_{l}+a_{c}+a_{t}+\gamma_{3}T^{*}-p_{t}I_{E}^{*}-p_{t}C_{L}^{*})\\ & +a_{11}a_{v},\\ {ℬ}_{3} & =[a_{11}a_{v}-\frac{\delta_{v}a_{i}\alpha_{i}(\Pi -I_{E}^{*})}{\left(1+\gamma_{1}T^{*}\right)}](a_{l}+a_{c}+a_{t}+\gamma_{3}T^{*}-p_{t}I_{E}^{*}-p_{t}C_{L}^{*}-a_{33})\\ & +(a_{v}-a_{11})[(a_{l}+a_{t})(a_{c}+\gamma_{3}T^{*})-\delta_{l}a_{c}\alpha_{l}\frac{C^{*}}{g+C^{*}}-p_{t}(I_{E}^{*}+C_{L}^{*})(a_{l}+a_{c}+\gamma_{3}T^{*})\\ & +p_{t}\gamma_{3}T^{*}C_{L}^{*}-a_{33}(a_{l}+a_{c}+a_{t}+\gamma_{3}T^{*}-p_{t}I_{E}^{*}-p_{t}C_{L}^{*})]+a_{l}p_{t}\gamma_{3}T^{*}C_{L}^{*}+a_{t}a_{l}(a_{c}+\gamma_{3}T^{*})\\ & -p_{t}a_{l}(I_{E}^{*}+C_{L}^{*})(a_{c}+\gamma_{3}T^{*})+p_{t}\delta_{l}a_{c}\alpha_{l}\frac{C^{*}(I_{E}^{*}+C_{L}^{*})}{g+C^{*}}+\delta_{v}\delta_{l}\frac{a_{i}\alpha_{i}\alpha_{l}^{2}a_{c}gC^{*}L^{*}(\pi -I_{E}^{*})}{\left(1+\gamma_{1}T^{*}\right)}\\ & \frac{\left(a_{t}-p_{t}I_{E}^{*}-p_{t}C_{L}^{*}\right)}{(g+C^{*}{)}^{2}}-a_{33}[a_{l}(a_{c}+\gamma_{3}T^{*})-\delta_{l}a_{c}\alpha_{l}\frac{C^{*}}{g+C^{*}}+(a_{t}-p_{t}I_{E}^{*}-p_{t}C_{L}^{*})\\ & (a_{l}+a_{c}+\gamma_{3}T^{*})+p_{t}\gamma_{3}T^{*}C_{L}^{*}]+(a_{v}-a_{11}-a_{33})\delta_{v}\delta_{l}\frac{a_{i}\alpha_{i}\alpha_{l}^{2}a_{c}gC^{*}L^{*}(\pi -I_{E}^{*})}{(1+\gamma_{1}T^{*})(g+C^{*}{)}^{2}}-p_{t}T^{*}, \end{array}$$

$$\begin{array}{cc} {ℬ}_{4} & =\delta_{l}a_{c}\alpha_{l}\frac{C^{*}}{g+C^{*}}-a_{l}(a_{c}+\gamma_{3}T^{*})+(p_{t}I_{E}^{*}+p_{t}C_{L}^{*}-a_{t})(a_{l}+a_{c}+\gamma_{3}T^{*})\\ & -\delta_{v}\delta_{l}\frac{a_{i}\alpha_{i}\alpha_{l}^{2}a_{c}gC^{*}L^{*}(\pi -I_{E}^{*})}{(1+\gamma_{1}T^{*})(g+C^{*}{)}^{2}}-p_{t}\gamma_{3}T^{*}C_{L}^{*}+a_{33}(1-a_{11}a_{v})(a_{l}+a_{c}+a_{t}+\gamma_{3}T^{*}-p_{t}I_{E}^{*}\\ & -p_{t}C_{L}^{*})+(a_{v}-a_{11}+a_{33})[a_{l}p_{t}\gamma_{3}T^{*}C_{L}^{*}+a_{l}(a_{t}-p_{t}I_{E}^{*}-p_{t}C_{L}^{*})(a_{c}+\gamma_{3}T^{*})\\ & +\delta_{v}\delta_{l}\frac{a_{i}\alpha_{i}\alpha_{l}^{2}a_{c}gC^{*}L^{*}(\pi -I_{E}^{*})}{\left(1+\gamma_{1}T^{*}\right)}\frac{\left(a_{t}-p_{t}I_{E}^{*}-p_{t}C_{L}^{*}\right)}{(g+C^{*}{)}^{2}}+p_{t}\delta_{l}a_{c}\alpha_{l}C^{*}\frac{I_{E}^{*}+C_{L}^{*}}{g+C^{*}}]\\ & +[a_{11}a_{v}-a_{33}(a_{v}-a_{11})][a_{l}(a_{c}+\gamma_{3}T^{*})-\delta_{l}a_{c}\alpha_{l}\frac{C^{*}}{g+C^{*}}-p_{t}(I_{E}^{*}+C^{*})(a_{l}+a_{c}+\gamma_{3}T^{*})\\ & +a_{t}(a_{l}+a_{c}+\gamma_{3}T^{*})+\delta_{v}\delta_{l}\frac{a_{i}\alpha_{i}\alpha_{l}^{2}a_{c}gC^{*}L^{*}(\pi -I_{E}^{*})}{(1+\gamma_{1}T^{*})(g+C^{*}{)}^{2}}+p_{t}\gamma_{3}T^{*}C_{L}^{*}]-a_{16}\beta_{c}\delta_{l}a_{c}\frac{\alpha_{l}gL^{*}}{(g+C^{*}{)}^{2}}\\ & -a_{l}-a_{c}-\gamma_{3}T^{*}+a_{33},\\ {ℬ}_{5} & =(a_{33}-a_{33}a_{11}a_{v})[a_{l}(a_{c}+\gamma_{3}T^{*})-\delta_{l}a_{c}\alpha_{l}\frac{C^{*}}{g+C^{*}}-p_{t}(I_{E}^{*}+C^{*})(a_{l}+a_{c}+\gamma_{3}T^{*})\\ & +a_{t}(a_{l}+a_{c}+\gamma_{3}T^{*})+\delta_{v}\delta_{l}\frac{a_{i}\alpha_{i}\alpha_{l}^{2}a_{c}gC^{*}L^{*}(\pi -I_{E}^{*})}{(1+\gamma_{1}T^{*})(g+C^{*}{)}^{2}}+p_{t}\gamma_{3}T^{*}C_{L}^{*}]+(a_{33}a_{v}-a_{33}a_{11}\\ & +a_{11}a_{v}-1)[a_{l}p_{t}\gamma_{3}T^{*}C_{L}^{*}+a_{t}a_{l}(a_{c}+\gamma_{3}T^{*})-p_{t}a_{l}(a_{c}+\gamma_{3}T^{*})(I_{E}^{*}+C_{L}^{*})\\ & +p_{t}\delta_{l}a_{c}\alpha_{l}C^{*}\frac{I_{E}^{*}+C_{L}^{*}}{g+C^{*}}+\delta_{v}\delta_{l}\frac{a_{i}\alpha_{i}\alpha_{l}^{2}a_{c}gC^{*}L^{*}(\pi -I_{E}^{*})}{\left(1+\gamma_{1}T^{*}\right)}\frac{\left(a_{t}-p_{t}I_{E}^{*}-p_{t}C_{L}^{*}\right)}{(g+C^{*}{)}^{2}}]\\ & -a_{16}\beta_{c}\delta_{l}a_{c}\frac{\alpha_{l}gL^{*}(a_{v}+a_{l})}{(g+C^{*}{)}^{2}}-a_{l}(a_{c}+\gamma_{3}T^{*})-a_{c}\delta_{l}\alpha_{l}\frac{C^{*}}{g+C^{*}}+a_{33}(a_{l}+a_{c}+\gamma_{3}T^{*}),\\ {ℬ}_{6} & =(a_{33}+a_{33}a_{11}a_{v})[a_{t}a_{l}(a_{c}+\gamma_{3}T^{*})-p_{t}a_{l}(I_{E}^{*}+C^{*})(a_{c}+\gamma_{3}T^{*})\\ & +\delta_{v}\delta_{l}\frac{a_{i}\alpha_{i}\alpha_{l}^{2}a_{c}gC^{*}L^{*}(\pi -I_{E}^{*})}{\left(1+\gamma_{1}T^{*}\right)}\frac{\left(a_{t}-p_{t}I_{E}^{*}-p_{t}C_{L}^{*}\right)}{(g+C^{*}{)}^{2}}+p_{t}\delta_{l}a_{c}\alpha_{l}C^{*}\frac{I_{E}^{*}+C_{L}^{*}}{g+C^{*}}\\ & +a_{l}p_{t}\gamma_{3}T^{*}C_{L}^{*}]-a_{16}\beta_{c}\delta_{l}a_{c}a_{v}a_{l}\frac{\alpha_{l}gL^{*}}{(g+C^{*}{)}^{2}}+a_{33}a_{l}(a_{c}+\gamma_{3}T^{*})-a_{33}a_{c}\delta_{l}\alpha_{l}\frac{C^{*}}{g+C^{*}}\\ & +\alpha_{l}^{2}gL^{*}\frac{C^{*}}{(g+C^{*}{)}^{3}}. \end{array}$$

Since the characteristic equation is of sixth order with analytically intractable roots, the Routh-Hurwitz criteria provide an effective approach to determine local stability without explicitly computing eigenvalues. Thus, in view of the discussions made above and using the Routh-Hurwitz criteria [41,42], we can state the following theorem.

**Theorem 5.** *The local asymptotic stability of the system (1) in the neighborhood of the endemic equilibrium point (EEP)*, $E_{n}=(I_{E}^{*},V^{*},C^{*},C_{L}^{*},L^{*},T^{*})$*, is ensured when the following conditions are satisfied:*


$$\begin{array}{cc} & {ℬ}_{1}>0,\;{ℬ}_{1}{ℬ}_{2}-{ℬ}_{3}>0,\;{ℬ}_{1}{ℬ}_{2}{ℬ}_{3}+{ℬ}_{1}{ℬ}_{5}>{ℬ}_{3}^{2}+{ℬ}_{1}^{2}{ℬ}_{4}\;\text{and}\;{ℬ}_{1}^{2}{ℬ}_{2}{ℬ}_{3}{ℬ}_{4}+{ℬ}_{1}{ℬ}_{2}{ℬ}_{3}{ℬ}_{5}\\ & +{ℬ}_{1}^{3}{ℬ}_{2}{ℬ}_{6}+2{ℬ}_{1}^{2}{ℬ}_{4}{ℬ}_{5}>{ℬ}_{1}{ℬ}_{4}{ℬ}_{3}^{2}+{ℬ}_{1}^{3}{ℬ}_{4}^{2}+{ℬ}_{1}{ℬ}_{5}^{2}+{ℬ}_{1}^{2}{ℬ}_{2}^{2}{ℬ}_{5}+{ℬ}_{1}^{2}{ℬ}_{3}{ℬ}_{6}. \end{array}$$

**Remark 4.** *The conditions in Theorem 5 ensure that perturbations around the endemic equilibrium decay over time, which indicates the persistence of HPV infection, cancer cells, and oncolytic viruses at stable levels within the host. Biologically, this corresponds to a chronic disease state under sustained viral and immune interactions.*

## 4 Sensitivity analysis

In complex nonlinear biological systems, model outcomes often depend sensitively on multiple parameters whose values may be uncertain or subject to biological variability. To identify the key parameters governing the dynamics of HPV-induced cervical cancer under oncolytic virotherapy, we perform a global sensitivity analysis using the Extended Fourier Amplitude Sensitivity Test (eFAST) [43,44]. This method quantifies both the individual effects of parameters and their higher-order interaction effects on model outputs, providing a robust assessment of parameter influence across the entire admissible parameter space. From a biological perspective, this analysis helps determine which processes such as viral replication, immune response, or cancer cell proliferation, most strongly influence disease outcomes and therapeutic efficacy. The in detail algorithm describing the global sensitivity analysis using eFAST is provided in Algorithm 1.

**Algorithm 1 Algorithm for global sensitivity analysis utilizing Extended Fourier Amplitude Sensitivity Test (eFAST)**

**Input:** Deterministic model $Y=f(X_{1},...,X_{k})$; bounds $\left[X_{i}^{\min},X_{i}^{\max}\right]$; base sample size *N* (even); replicates *M*; harmonic multiplier *p*; distinct integer frequencies $\left\{\omega_{i}\right\}$ with $p\omega_{i}<N/2$.

**Output:** First-order indices $S_{i}$ and total-order indices $S_{T,i}$ for i=1,...,k.

1: **(Sample)** For each replicate r=1,...,M choose random phase $\phi_{r}\in [0,2\pi )$ and for j=1,...,N set


$$s_{j}=\frac{2\pi j}{N},\;u_{i,j}^{\left(r\right)}=\frac{1}{2}+\frac{1}{\pi }\arcsin\;(\sin(\omega_{i}s_{j}+\phi_{r})),$$

then map $u_{i,j}^{\left(r\right)}$ to parameter values by the chosen prior (e.g., uniform):


$$X_{i}^{\left(r\right)}(s_{j})=X_{i}^{\min}+u_{i,j}^{\left(r\right)}(X_{i}^{\max}-X_{i}^{\min}).$$

2: **(Model eval)** For each replicate *r* and *j*, evaluate $Y_{j}^{\left(r\right)}=f(X_{1}^{\left(r\right)}(s_{j}),...,X_{k}^{\left(r\right)}(s_{j}))$.

3: **(Spectral decomposition)** For each replicate compute the DFT coefficients


$${\hat{Y}}_{n}^{\left(r\right)}=\frac{1}{N}\sum_{j=1}^{N}Y_{j}^{\left(r\right)}e^{-ins_{j}},\;n=0,...,N-1,$$

and spectral power $P_{n}^{\left(r\right)}=2|{\hat{Y}}_{n}^{\left(r\right)}{|}^{2}$ for n=1,...,N/2−1. Total variance: $D^{\left(r\right)}=\sum_{n=1}^{N/2-1}P_{n}^{\left(r\right)}$.

4: **(First-order** $S_{i}$**)** Assign main-effect band $B_{i}=\{\omega_{i},...,p\omega_{i}\}$. Compute


$$D_{i}^{\left(r\right)}=\sum_{n\in B_{i}}P_{n}^{\left(r\right)},\;S_{i}^{\left(r\right)}=\frac{D_{i}^{\left(r\right)}}{D^{\left(r\right)}}.$$

5: **(Total-order** $S_{T,i}$**)** Using the complementary spectral method (fast):


$$D_{\sim i}^{\left(r\right)}=\sum_{n\notin B_{i}}P_{n}^{\left(r\right)},\;S_{T,i}^{\left(r\right)}=1-\frac{D_{\sim i}^{\left(r\right)}}{D^{\left(r\right)}}.$$

(Alternatively, use the resampling approach by randomizing $X_{i}$ for a robust $S_{T,i}$.)

6: **(Aggregate)** Average over replicates:


$${\bar{S}}_{i}=\frac{1}{M}\sum_{r=1}^{M}S_{i}^{\left(r\right)},\;{\bar{S}}_{T,i}=\frac{1}{M}\sum_{r=1}^{M}S_{T,i}^{\left(r\right)}.$$

Report standard errors (or bootstrap percentiles) across replicates.

7: **(Diagnostics)** Verify $p\omega_{\max}<N/2$, check spectrum for aliasing, test convergence by increasing *N* or *M*, and confirm ${\bar{S}}_{i},{\bar{S}}_{T,i}\in [0,1]$.

8: **return** $\{{\bar{S}}_{i},{\bar{S}}_{T,i}{\}}_{i=1}^{k}$ with uncertainty estimates.

## 5 System equipped with control therapeutic approach

Optimal control provides a powerful mathematical framework for guiding the dynamics of biological systems [45,46]. In the context of treatment, such problems involve determining time-dependent control functions over a specified period. Here, we formulate an optimal control problem (13) based on the proposed mathematical model (1), incorporating the effects of both chemotherapeutic drugs and virotherapy. To track the progression of the chemotherapeutic drug within the system, we introduce a state variable *M*(*t*), which represents the drug concentration over time. The control input $u_{m}(t)$ denotes the dosage of chemotherapy administered, while $\gamma_{4}$ characterizes the decay rate of the drug during its administration period. Control $u_{m}$ plays a crucial role in reducing the production of cancer cells in the cervix. The drug affects all cell types, although the magnitude of its cytotoxic effects varies across them. This differential cytotoxic effect is modeled by the function $h_{r}(M)=d_{r}\left(1-e^{-\frac{M(t)}{M_{0}}}\right)$, *r* = *i*, *c*, *l*, *t*. Accordingly, the impact of the drug on each cell type is expressed as $-h_{r}(M)X$, where $X\in \{I_{E},C,C_{L},T\}$ represents the corresponding cell populations. This formulation captures the distinct effects of chemotherapy on different ce*l*l *t*ypes through varying values of $h_{r}(M)$. The control $u_{l}(t)$ denotes the dose of virotherapy that infects and eradicates cancer cells. To reduce the risk of side effects associated with high-dose treatments, the objective is to minimize the total amount of virotherapy and chemotherapy administered while effectively reducing cancer and infected cancer cell populations. Here, $0\leq u_{l}(t)\leq 1$ and $0\leq u_{m}(t)\leq 1$, for $t\in [0,T_{f}]$. Accordingly, the set of admissible controls is defined as


$$\begin{array}{cc} 𝔸 & =\{(u_{l},u_{m}):u_{l},u_{m}\text{ are measurable functions with }0\leq u_{l}(t)\leq 1,\;0\leq u_{m}(t)\leq 1,\;\\ & t\in [0,T_{f}]\}. \end{array}$$ (12)

The state system under the action of the control functions $u_{l}(t)$ and $u_{m}(t)$ is given by


$$\begin{array}{cc} \frac{dI_{E}}{dt} & =\frac{\alpha_{i}V(\Pi -I_{E})}{1+\gamma_{1}T}+p_{i}I_{E}-a_{i}I_{E}-\beta_{c}I_{E}-\gamma_{2}TI_{E}-d_{i}\left(1-e^{-\frac{M}{M_{0}}}\right)I_{E},\\ \frac{dV}{dt} & =\delta_{v}a_{i}I_{E}-a_{v}V,\\ \frac{dC}{dt} & =\beta_{c}I_{E}+p_{c}C(1-kC)-\frac{\alpha_{l}LC}{g+C}-d_{c}\left(1-e^{-\frac{M}{M_{0}}}\right)C,\\ \frac{dC_{L}}{dt} & =\frac{\alpha_{l}LC}{g+C}-a_{c}C_{L}-\gamma_{3}TC_{L}-d_{l}\left(1-e^{-\frac{M}{M_{0}}}\right)C_{L},\\ \frac{dL}{dt} & =u_{l}(t)+\delta_{l}a_{c}C_{L}-a_{l}L,\\ \frac{dT}{dt} & =(p_{t}I_{E}+p_{t}C_{L})T-a_{t}T-d_{t}\left(1-e^{-\frac{M}{M_{0}}}\right)T,\\ \frac{dM}{dt} & =u_{m}(t)-\gamma_{4}M \end{array}$$ (13)

and the initial conditions are given as


$$\begin{array}{cc} I_{E}(0) & =I_{E_{0}}>0,V(0)=V_{0}>0,C(0)=C_{0}\geq 0,C_{L}(0)=C_{L_{0}}\geq 0,\\ L(0) & =L_{0}\geq 0,T(0)=T_{0}\geq 0,\text{ and }M(0)=M_{0}>0. \end{array}$$ (14)

We define the objective functional as


$$\begin{array}{c} 𝒥(u_{l},u_{m})=\int_{0}^{T_{f}}\left[C(t)+C_{L}(t)+\frac{1}{2}\left(\omega_{1}u_{l}^{2}(t)+\omega_{2}u_{m}^{2}(t)\right)\right]dt. \end{array}$$ (15)

Here, $\omega_{1}$ and $\omega_{2}$ are positive weight parameters that balance the relative cost of administering virotherapy and chemotherapy against the objective of reducing the cancer and infected cancer cell populations. The first and second terms on the right-hand side of (15) represent cancerous cells and infected cancerous cells, respectively, while the third and fourth terms account for the effectiveness of the drugs.

Under the adopted baseline parameter set, the proposed mathematical model (i.e., the uncontrolled system (1)) admits a unique biologically feasible endemic equilibrium, as established in Section 3.3.1. Thus, within this baseline parameter regime, the model does not exhibit multiple biologically feasible endemic equilibria. Accordingly, the subsequent optimal control analysis for the controlled system (13) is performed with respect to this parameter regime.

Our objective is to determine an optimal control pair $\left(u_{l}^{*}(t),u_{m}^{*}(t)\right)$ such that


$$\begin{array}{c} 𝒥(u_{l}^{*},u_{m}^{*})=\min\left\{𝒥(u_{l},u_{m}):(u_{l},u_{m})\in 𝔸\right\}. \end{array}$$ (16)

Before characterizing the optimal control, We first address the well-posedness of the optimal control problem by establishing boundedness of solutions and existence of an optimal control in the next subsection.

### 5.1 Boundedness and existence

We examine the boundedness and existence of the optimal control problem (13) with initial condition (14) using the results of Fleming & Rishel, and Lukes [45,47] in the following theorem.

**Theorem 6.** *Let* $X^{*}(t)$ *denote the optimal state trajectory of system (13) associated with the optimal controls* $\left(u_{l}^{*}(t),u_{m}^{*}(t)\right)$. *For the optimal control problem (13) with initial condition (14) satisfying (16), there exists an optimal solution* $\left(X^{*}(t),u_{l}^{*}(t),u_{m}^{*}(t))\in {𝔸}^{1,\infty }([0,T_{f}],{\mathbb{R}}_{+}^{7})\times {𝕋}^{\infty }([0,T_{f}],{\mathbb{R}}_{+}^{2}\right)$ *if*

1 ***(Admissible Set Conditions)*** *The set* 𝔸 *is nonempty, closed, and convex.*
2 ***(System Dynamics Regularity)*** *The right-hand side of the system of equations in (13) is linearly bounded in terms of the state and control variables.*
3 ***(Convexity of the Cost Functional)*** *The integrand of the cost functional* $𝒥(u_{l},u_{m})$,

$$\begin{array}{c} f(u_{l},u_{m})=C(t)+C_{L}(t)+\frac{1}{2}\left[\omega_{1}u_{l}^{2}(t)+\omega_{2}u_{m}^{2}(t)\right], \end{array}$$

*is convex over the admissible set* 𝔸.
4 ***(Coercivity Condition)*** *There exist constants* ω>0 *and* θ>0 *such that*

$$\begin{array}{c} f(u_{l},u_{m})\geq \frac{1}{2}\omega \left(u_{l}^{2}+u_{m}^{2}\right)-\theta , \end{array}$$

*for all* $(u_{l},u_{m})\in 𝔸$.

*Here,* ${𝔸}^{1,\infty }([0,T_{f}],{\mathbb{R}}_{+}^{7})$ *denotes the space of absolutely continuous state trajectories with essentially bounded derivatives, and* ${𝕋}^{\infty }([0,T_{f}],{\mathbb{R}}_{+}^{2})$ *denotes the space of essentially bounded measurable control functions on* $\left[0,T_{f}\right]$.

*Proof.* Let $N(t)=\gamma_{3}p_{t}I_{E}+V+\gamma_{2}p_{t}C+\gamma_{2}p_{t}C_{L}+\gamma_{1}\gamma_{2}T+L$.

From Theorem (2), we have that all state variables are uniformly bounded, i.e.,


$$\begin{array}{c} I_{E},V,C,C_{L},L,T\leq \frac{\gamma_{2}p_{t}p_{c}}{nk}. \end{array}$$

From system (13), we have


$$\begin{array}{c} \left\{ \begin{array}{ccc} \frac{dI_{E}}{dt} & < & \alpha_{i}\pi V+p_{i}I_{E},\\ \frac{dV}{dt} & < & \delta_{v}a_{i}I_{E},\\ \frac{dC}{dt} & < & \beta_{c}I_{E}+p_{c}C,\\ \frac{dC_{L}}{dt} & < & \frac{\gamma_{2}p_{t}p_{c}}{nk}L,\\ \frac{dL}{dt} & < & \delta_{l}a_{c}C_{L}+u_{l}(t),\\ \frac{dT}{dt} & < & \frac{2\gamma_{2}p_{t}^{2}p_{c}}{nk}T,\\ \frac{dM}{dt} & < & u_{m}(t). \end{array}\right. \end{array}$$ (17)

We represent system (17) in matrix form as follows


$$\begin{array}{c} (\begin{array}{c} \frac{dI_{E}}{dt}\\ \frac{dV}{dt}\\ \frac{dC}{dt}\\ \frac{dC_{L}}{dt}\\ \frac{dL}{dt}\\ \frac{dT}{dt}\\ \frac{dM}{dt} \end{array})<(\begin{array}{ccccccc} p_{i} & \alpha_{i}\pi & 0 & 0 & 0 & 0 & 0\\ \delta_{v}a_{i} & 0 & 0 & 0 & 0 & 0 & 0\\ \beta_{c} & 0 & p_{c} & 0 & 0 & 0 & 0\\ 0 & 0 & 0 & 0 & \frac{\gamma_{2}p_{t}p_{c}}{nk} & 0 & 0\\ 0 & 0 & 0 & \delta_{l}a_{c} & 0 & 0 & 0\\ 0 & 0 & 0 & 0 & 0 & \frac{2\gamma_{2}p_{t}^{2}p_{c}}{nk} & 0\\ 0 & 0 & 0 & 0 & 0 & 0 & 0 \end{array})(\begin{array}{c} I_{E}\\ V\\ C\\ C_{L}\\ L\\ T\\ M \end{array})+(\begin{array}{c} 0\\ 0\\ 0\\ 0\\ u_{l}(t)\\ 0\\ u_{m}(t) \end{array}). \end{array}$$ (18)

Since system (18) is linear with bounded coefficients and bounded inputs $u_{l}(t)$ and $u_{m}(t)$, it follows that all state trajectories of (13) remain bounded on $\left[0,T_{f}\right]$.

Using the definition of 𝔸, we conclude that 𝔸 is convex and closed.

The integrand of $𝒥(u_{l},u_{m})$ is


$$\begin{array}{c} f(u_{l},u_{m})=C(t)+C_{L}(t)+\frac{1}{2}\left[\omega_{1}u_{l}^{2}(t)+\omega_{2}u_{m}^{2}(t)\right] \end{array}$$

with $(u_{l},u_{m})\in 𝔸.$

It suffices to show the convexity of $f(u_{l},u_{m})$ on 𝔸, i.e., $f[(1-\rho )y_{1}+\rho y_{2}]\leq (1-\rho )f(y_{1})+\rho f(y_{2}),$ where $y_{1}=(u_{l_{1}},u_{m_{1}}),y_{2}=(u_{l_{2}},u_{m_{2}})\in 𝔸$ and 0≤ρ≤1.

Now,


$$\begin{array}{cc} & f\left[(1-\rho )y_{1}+\rho y_{2}\right]-\left[(1-\rho )f(y_{1})+\rho f(y_{2})\right]\\ & =\frac{\omega_{1}}{2}{\left[(1-\rho )u_{l_{1}}+\rho u_{l_{2}}\right]}^{2}+\frac{\omega_{2}}{2}{\left[(1-\rho )u_{m_{1}}+\rho u_{m_{2}}\right]}^{2}-\frac{1}{2}(1-\rho )\left[\omega_{1}u_{l_{1}}^{2}+\omega_{2}u_{m_{1}}^{2}\right]\\ & -\frac{1}{2}\rho \left[\omega_{1}u_{l_{2}}^{2}+\omega_{2}u_{m_{2}}^{2}\right]\\ & =\frac{\omega_{1}}{2}\rho (\rho -1)(u_{l_{1}}-u_{l_{2}}{)}^{2}+\frac{\omega_{2}}{2}\rho (\rho -1)(u_{m_{1}}-u_{m_{2}}{)}^{2}\\ & \leq 0,\;\text{since}\;0\leq \rho \leq 1, \end{array}$$

which proves the convexity of $f(u_{l},u_{m})$ on 𝔸.

Again,


$$\begin{array}{cc} f(u_{l},u_{m}) & =C(t)+C_{L}(t)+\frac{1}{2}[\omega_{1}u_{l}^{2}(t)+\omega_{2}u_{m}^{2}(t)],\\ & \geq \frac{1}{2}[\omega_{1}u_{l}^{2}(t)+\omega_{2}u_{m}^{2}(t)]\\ & \geq \frac{1}{2}\rho (u_{l}^{2}+u_{m}^{2});\text{ where }\rho =\min\{\omega_{1},\omega_{2}\}\\ & \geq \frac{1}{2}\rho (u_{l}^{2}+u_{m}^{2})-\theta \text{ where }\theta >0. \end{array}$$

Hence, the objective functional $𝒥(u_{l},u_{m})$ is bounded below and attains its minimum over the admissible control set 𝔸. Therefore, there exists an optimal control pair $\left(u_{l}^{*}(t),u_{m}^{*}(t)\right)$ satisfying the control problem (13)–(16). □

### 5.2 Characterization of optimal control

Previously, we have established the conditions for the existence of a solution to the optimal control problem(13). We now derive the necessary conditions for an optimal solution to the problem(13)-(15) using Pontryagin’s Maximum Principle. To solve the problem, we consider the Hamiltonian as


$$\begin{array}{cc} & ℋ(X(t),\xi (t),u_{l}(t),u_{m}(t))\\ & =C(t)+C_{L}(t)+\frac{1}{2}[\omega_{1}u_{l}^{2}(t)+\omega_{2}u_{m}^{2}(t)]\\ & +\xi_{1}[\frac{\alpha_{i}V(\Pi -I_{E})}{1+\gamma_{1}T}+p_{i}I_{E}-a_{i}I_{E}-\beta_{c}I_{E}-\gamma_{2}TI_{E}-d_{i}(1-e^{-\frac{M}{M_{0}}})I_{E}]\\ & +\xi_{2}[\delta_{v}a_{i}I_{E}-a_{v}V]+\xi_{3}[\beta_{c}I_{E}+p_{c}C(1-kC)-\frac{\alpha_{l}LC}{g+C}-d_{c}(1-e^{-\frac{M}{M_{0}}})C]\\ & +\xi_{4}[\frac{\alpha_{l}LC}{g+C}-a_{c}C_{L}-\gamma_{3}TC_{L}-d_{l}(1-e^{-\frac{M}{M_{0}}})C_{L}]+\xi_{5}[u_{l}(t)+\delta_{l}a_{c}C_{L}-a_{l}L]\\ & +\xi_{6}[(p_{t}I_{E}+p_{t}C_{L})T-a_{t}T-d_{t}(1-e^{-\frac{M}{M_{0}}})T]+\xi_{7}[u_{m}(t)-\gamma_{4}M], \end{array}$$

where $\xi =(\xi_{1},\xi_{2},\xi_{3},\xi_{4},\xi_{5},\xi_{6},\xi_{7})$ are adjoint variables.

Using the Hamiltonian formulation described above, Pontryagin’s Maximum Principle provides the necessary conditions that must be satisfied by an optimal control and the corresponding state trajectory. In particular, the principle yields the adjoint system, transversality conditions, and a pointwise minimization condition for the optimal controls. These conditions are summarized in the following theorem.

**Theorem 7.** *If* $\left(X^{*},u_{l}^{*},u_{m}^{*})\in {𝔸}^{1,\infty }([0,T_{f}],{\mathbb{R}}_{+}^{7})\times {𝕋}^{\infty }([0,T_{f}],{\mathbb{R}}_{+}^{2}\right)$ *be a solution of control problem (13) satisfying (14)-(15), then* ∃ *an adjoint* $\xi \in {𝒥}^{1,\infty }([0,T_{f}],{\mathbb{R}}_{+}^{7})$ *such that*

i) *The adjoint variables satisfy*

$$\begin{array}{c} \frac{d\xi }{dt}=-\frac{\partial ℋ}{\partial X} \end{array}$$

*with the transversality conditions*

$$\begin{array}{c} \xi_{i}(T_{f})=0,\;i=1,2,3,4,5,6,7. \end{array}$$

ii) *The optimal controls* $u_{l}^{*}(t)$ *and* $u_{m}^{*}(t)$ *minimize the Hamiltonian almost everywhere on* $\left[0,T_{f}\right]$*, i.e.,*

$$\begin{array}{c} ℋ(X^{*}(t),\xi (t),u_{l}^{*}(t),u_{m}^{*}(t))=\min_{0\leq u_{l}\leq 1,\;0\leq u_{m}\leq 1}ℋ(X^{*}(t),\xi (t),u_{l},u_{m}), \end{array}$$

*for a.e.* $t\in [0,T_{f}]$.

*Proof.* Using Pontryagin Maximum Principle [46], we have


$$\begin{array}{cc} \frac{d\xi_{1}}{dt} & =-\frac{\partial ℋ}{\partial I_{E}}=\xi_{1}\left[\frac{\alpha_{i}V}{1+\gamma_{1}T}-p_{i}+a_{i}+\beta_{c}+\gamma_{2}T+d_{i}(1-e^{-\frac{M}{M_{0}}})\right]-\xi_{2}\delta_{v}a_{i}-\xi_{3}\beta_{c}-\xi_{6}p_{t}T,\\ \frac{d\xi_{2}}{dt} & =-\frac{\partial ℋ}{\partial V}=-\xi_{1}\frac{\alpha_{i}(\Pi -I_{E})}{1+\gamma_{1}T}+\xi_{2}a_{v},\\ \frac{d\xi_{3}}{dt} & =-\frac{\partial ℋ}{\partial C}=\xi_{3}\left[2p_{c}kC-p_{c}+\frac{g\alpha_{l}L}{(g+C{)}^{2}}+d_{c}(1-e^{-\frac{M}{M_{0}}})\right]-\xi_{4}\frac{g\alpha_{l}L}{(g+C{)}^{2}}-1,\\ \frac{d\xi_{4}}{dt} & =-\frac{\partial ℋ}{\partial C_{L}}=\xi_{4}\left[a_{c}+\gamma_{3}T+d_{l}(1-e^{-\frac{M}{M_{0}}})\right]-\xi_{5}\delta_{l}a_{c}-\xi_{6}p_{t}T-1,\\ \frac{d\xi_{5}}{dt} & =-\frac{\partial ℋ}{\partial L}=\xi_{3}\frac{\alpha_{l}C}{g+C}-\xi_{4}\frac{\alpha_{l}C}{g+C}+\xi_{5}a_{l},\\ \frac{d\xi_{6}}{dt} & =-\frac{\partial ℋ}{\partial T}=\xi_{1}\left[\frac{\gamma_{1}\alpha_{i}V(\Pi -I_{E})}{(1+\gamma_{1}T{)}^{2}}+\gamma_{2}I_{E}\right]+\xi_{4}\gamma_{3}C_{L}-\xi_{6}\left[p_{t}I_{E}+p_{t}C_{L}-a_{t}-d_{t}(1-e^{-\frac{M}{M_{0}}})\right],\\ \frac{d\xi_{7}}{dt} & =-\frac{\partial ℋ}{\partial M}=\xi_{1}d_{i}I_{E}\frac{1}{M_{0}}e^{-\frac{M}{M_{0}}}+\xi_{3}d_{c}C\frac{1}{M_{0}}e^{-\frac{M}{M_{0}}}+\xi_{4}d_{l}C_{L}\frac{1}{M_{0}}e^{-\frac{M}{M_{0}}}+\xi_{6}d_{t}T\frac{1}{M_{0}}e^{-\frac{M}{M_{0}}}+\xi_{7}\gamma_{4}. \end{array}$$

The transversality conditions are


$$\begin{array}{c} \xi_{i}(T_{f})=0\;\text{for}\;i=1,2,...,7. \end{array}$$

Using the optimality conditions of $u_{l}$ and $u_{m}$, we find


$$\begin{array}{cc} \frac{\partial ℋ}{\partial u_{l}}{|}_{u_{l}=u_{l}^{*}(t)} & =0⟹u_{l}^{*}=-\frac{\xi_{5}}{\omega_{1}},\\ \frac{\partial ℋ}{\partial u_{m}}{|}_{u_{m}=u_{m}^{*}(t)} & =0⟹u_{m}^{*}=-\frac{\xi_{7}}{\omega_{2}}. \end{array}$$

Since the admissible controls are bounded between 0 and 1, the optimal controls are obtained by projecting the unconstrained controls onto the admissible set. Consequently, the optimal controls are given by


$$\begin{array}{c} u_{l}^{*}(t)=\left\{ \begin{array}{c} 0,\;\text{if}\;u_{l}^{*}\leq 0\\ -\frac{\xi_{5}}{\omega_{1}},\;\text{if}\;0<u_{l}^{*}<1\\ 1,\;\text{if}\;u_{l}^{*}\geq 1 \end{array}\right. \end{array}$$ (19)

and


$$\begin{array}{c} u_{m}^{*}(t)=\left\{ \begin{array}{c} 0,\;\text{if}\;u_{m}^{*}\leq 0\\ -\frac{\xi_{7}}{\omega_{2}},\;\text{if}\;0<u_{m}^{*}<1\\ 1,\;\text{if}\;u_{m}^{*}\geq 1. \end{array}\right. \end{array}$$ (20)

Therefore,


$$\begin{array}{c} u_{l}^{*}(t)=\min\left\{\max\left\{0,-\frac{\xi_{5}}{\omega_{1}}\right\},1\right\},\;u_{m}^{*}(t)=\min\left\{\max\left\{0,-\frac{\xi_{7}}{\omega_{2}}\right\},1\right\}. \end{array}$$

□

## 6 Numerical simulations and biological interpretation

In this present section, we have performed numerical simulations to investigate the nonlinear dynamics of cervical cancer progression described by the proposed model (1), (13) and to translate the analytical findings into biologically meaningful insights. The simulations capture the evolution of cervical cancer, immune responses, and viral interactions, thereby elucidating the mechanisms which governs the disease progression and the potential impact of therapeutic control strategies aimed at mitigating cervical cancer burden.

**Fig 2:**
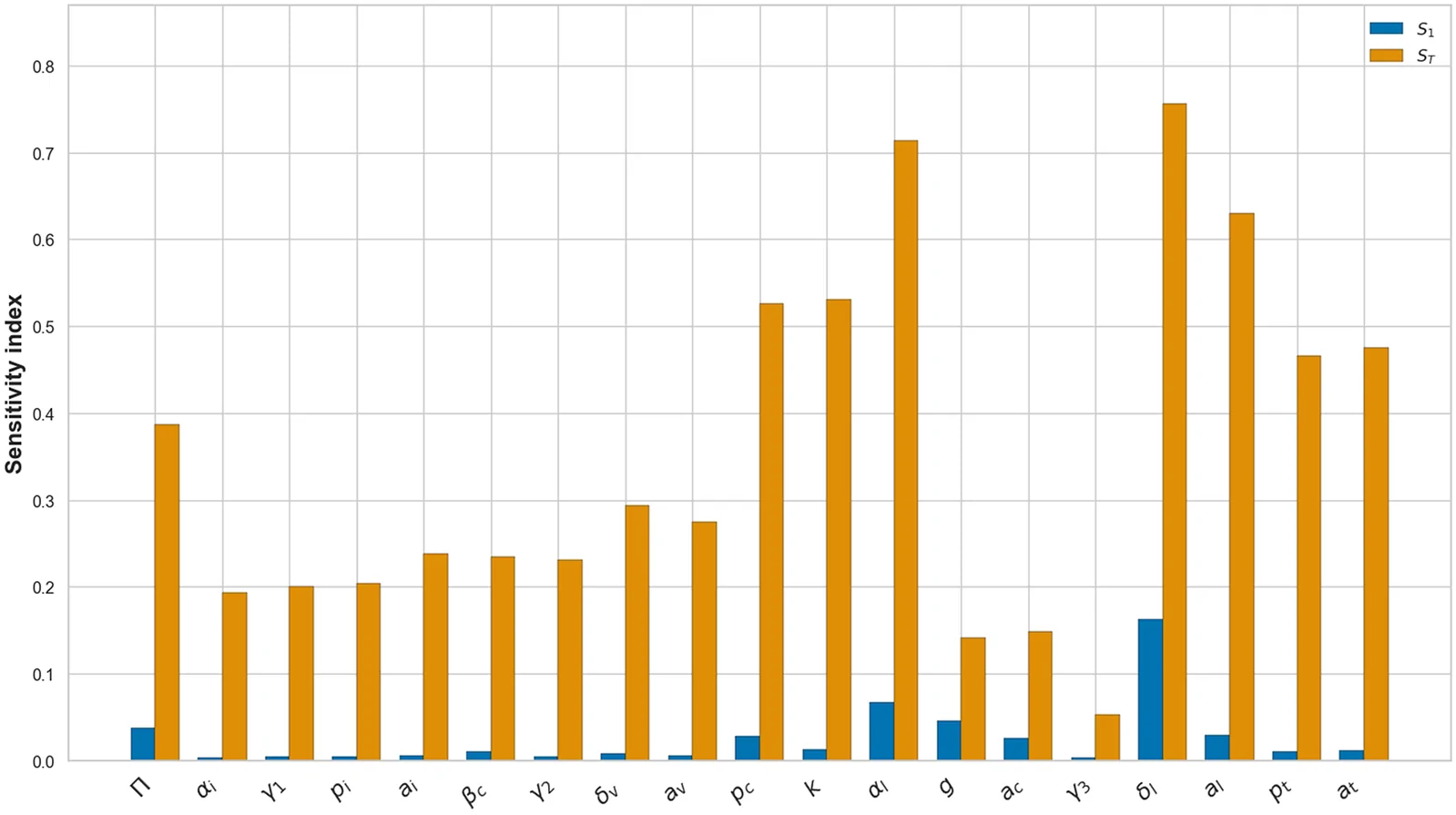
First-order (*S*_1_) and total-order ($S_{T}$) sensitivity indices of the cancer cell population *C*(*t*) with respect to key model parameters of system (1), computed using the extended Fourier Amplitude Sensitivity Test (eFAST) method. The first-order index *S*_1_ quantifies the direct contribution of each parameter to the variance of *C*(*t*), while the total-order index $S_{T}$ cap*t*ures both the individual effects and higher-order interaction effects among parameters. All selected parameters were varied simultaneously within biologically relevant ranges, while the remaining parameters were fixed at the baseline values. The parameter ranges (see Table 1) were chosen based on available literature and model assumptions to reflect physiologically plausible variability.

To quantify the relative importance of model parameters of system (1) on the cancer cell population, a global sensitivity analysis is performed using the extended Fourier Amplitude Sensitivity Test (eFAST), as described in the preceding Section, i.e., in Section 4. Fig 2 illustrates the first-order (*S*_1_) and total-order ($S_{T}$) sensitivity indices associated with the state variable *C*(*t*). The first-order index measures the direct effec*t* of individual parameters on the variance of *C*(*t*), whereas the total-order index accoun*t*s for both individual effects and all higher-order interactions. The results reveal that several parameters exhibit relatively small first-order sensitivity indices but significantly larger total-order indices, indicating that interaction effects dominate their overall influence on cancer cell dynamics. In particular, parameters such as the free OV virions bursting size ($\delta_{l}$), free OV virions clearance rate ($a_{l}$), inverse of the carrying capacity of the cancer cell population (*k*), and OV-induced infection rate of cancer cells ($\alpha_{l}$) demonstrate high total-order sensitivity. It suggests that therapeutic efficacy and cancer progression in the cervix are strongly governed by coupled biological mechanisms rather than isolated parameter effects. These findings highlight the importance of considering nonlinear interactions when designing and optimizing treatment strategies within the proposed model framework. Similar observations have also been reported in recent studies of oncolytic virotherapy [48], where viral infectivity, viral clearance, burst size, and tumor growth-limiting parameters were identified as key determinants of treatment outcome [49–51].

**Fig 3:**
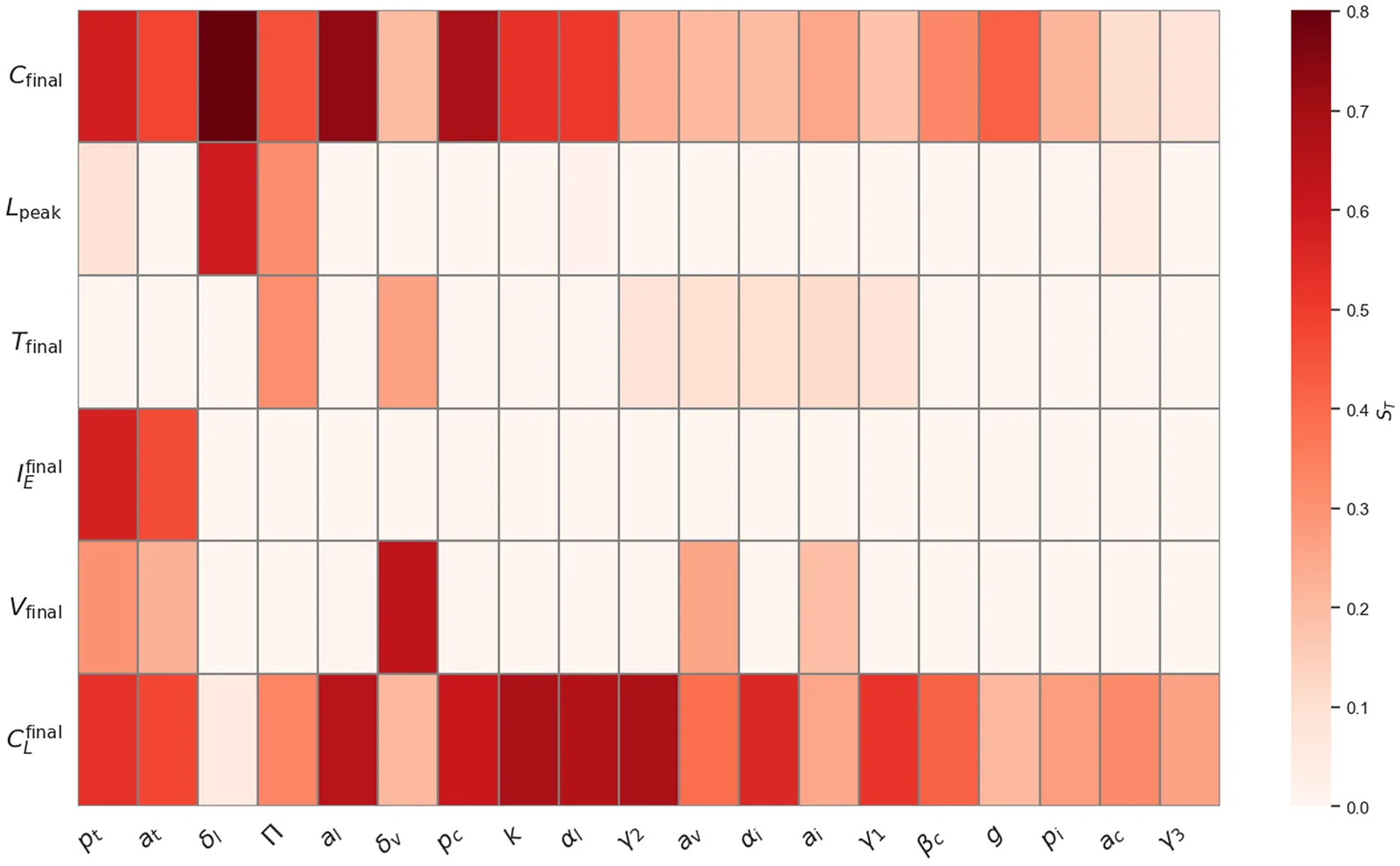
Demonstration of eFAST global sensitivity analysis heatmap of the cervical cancer-oncolytic virotherapy model denoted by system (1). The Figure illustrates the total-order sensitivity indices ($S_{T}$) of key model parameters on clinically and biologically relevant outcome variables. Color intensity reflects the relative contribution of each parameter to output variability, with darker shades indicating stronger influence as defined in the right-most column.

In Fig 3, a heatmap associated with the global sensitivity analysis utilizing the eFAST method is provided for system (1). The rows correspond to the final cancer cell burden (*C*_final_), peak oncolytic virus load (*L*_peak_), final cytotoxic T-lymphocyte population (*T*_final_), persistent HPV-infected epithelial cells ($I_{E}^{\text{final}}$), residual HPV viral load (*V*_final_), and infected cancer cells ($C_{L}^{\text{final}}$). The simulation represents the $S_{T}$ indices with respect to all the system parameters. The final cancer burden (*C*_final_) is most sensitive to the intrinsic cancer growth rate ($p_{c}$) and death rate ($a_{c}$), underscoring the net proliferation dynamics as the primary determinant of long-term tumor size. The peak oncolytic virus titer (*L*_peak_) is overwhelmingly governed by the viral burst size ($\delta_{l}$) and infection rate ($\alpha_{l}$), directly linking therapeutic potency to viral replication kinetics. The persistence of the adaptive immune response (*T*_final_) is primarily shaped by the CTL proliferation rate ($p_{t}$) and secondarily by the antigen supply from infected cells (influenced by $\beta_{c}$ and $\alpha_{l}$). Conversely, the clearance of the HPV infection (*I*_final_, *V*_final_) is most sensitive to the HPV production rate ($\delta_{v}$) and the CTL-mediated lytic ($\gamma_{2}$) and non-lytic ($\gamma_{1}$) effects. This structured sensitivity profile validates the model’s logic, clearly separating the key controllers for tumor growth, virotherapy dynamics, and immune response, thereby identifying high-leverage parameters ($\delta_{l}$, $\alpha_{l}$, $p_{t}$, $\gamma_{1,2}$) for potential therapeutic targeting.

**Fig 4:**
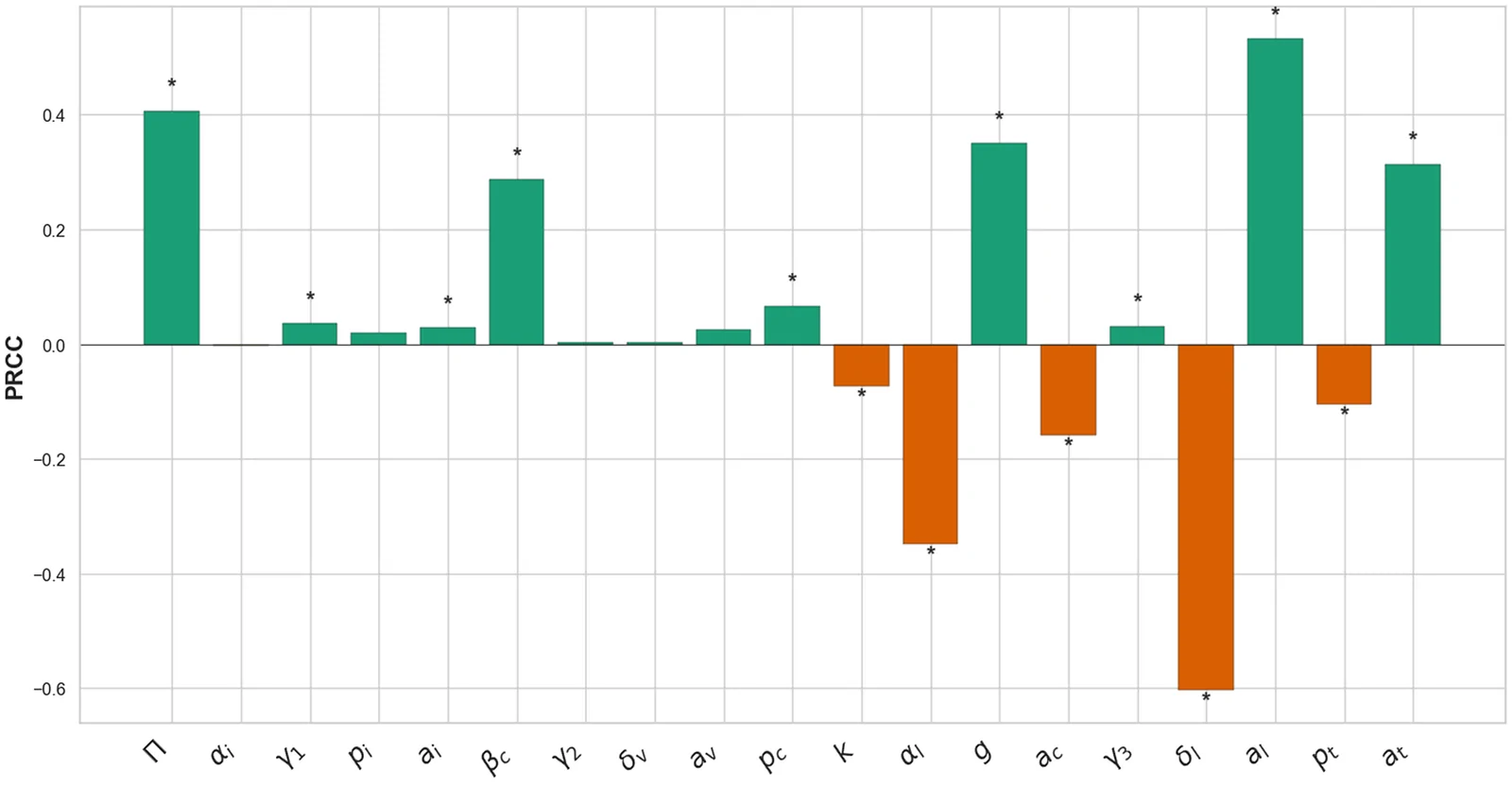
Partial Rank Correlation Coefficient (PRCC) analysis for the cancer cell population (*C*). The analysis identifies model parameters with a statistically significant (*p* < 0.05, denoted by *) monotonic influence on the final cancer cell burden. Parameters with positive (negative) PRCC values indicate a positive (inverse) correlation with the cancer cell density.

The monotonic influence of model parameters on the cancer cell population (*C*) is examined using Partial Rank Correlation Coefficient (PRCC) analysis, with significance determined at *p* < 0.05 with 95% confidence interval (Fig 4). The analysis reveals distinct promotive and inhibitory roles. It is clearly observed that the cancer cell population shows strongly positive correlation with the transformation rate of HPV-infected cells to cancer ($\beta_{c}$), the OV infection saturation constant (*g*), and the decay rates of the oncolytic virus ($a_{l}$) and CTLs ($a_{t}$). This indicates that slower immune decay, reduced viral infectivity (higher *g*), and a higher rate of malignant transformation all favor growth of cancer cell. Along with this, *C* is most negatively correlated with the OV infection rate ($\alpha_{l}$), the death rate of infected cancer cells ($a_{c}$), and the OV burst size ($\delta_{l}$).

**Fig 5:**
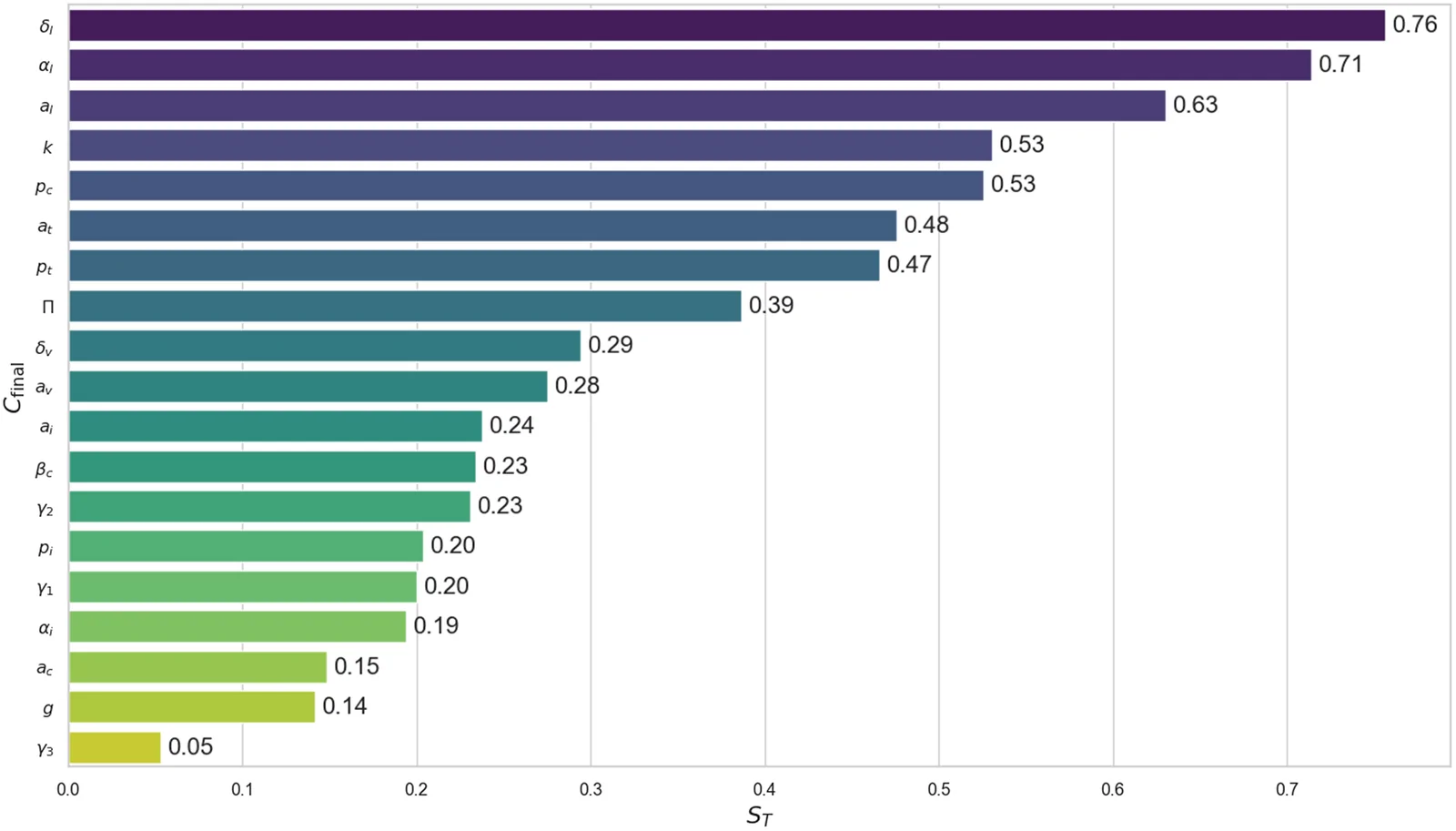
Rank-wise distribution of total-order sensitivity indices ($S_{T}$) of the parameters for system (1) from the eFAST analysis for the cancer cell compartment (*C*). The bars represent the $S_{T}$ values for each model parameter, ranked in descending order of influence on the variability of the cancer cell population.

The total-order sensitivity indices ($S_{T}$) for the cancer cell compartment (*C*) are ranked in descending order in Fig 5, providing a hierarchical view of parameter influence. The oncolytic virus (OV) clearance rate $a_{l}$ and burst size $\delta_{l}$ are the most significant. *k* and OV infection rate $\alpha_{l}$ follow closely while the CTL proliferation and decay rates ($p_{t}$, $a_{t}$) also hold substantial impacts. This ranking demonstrates that the variability in tumor burden is most sensitive to therapeutic viral properties, physical tumor constraints, and adaptive immune regulation.

**Fig 6:**
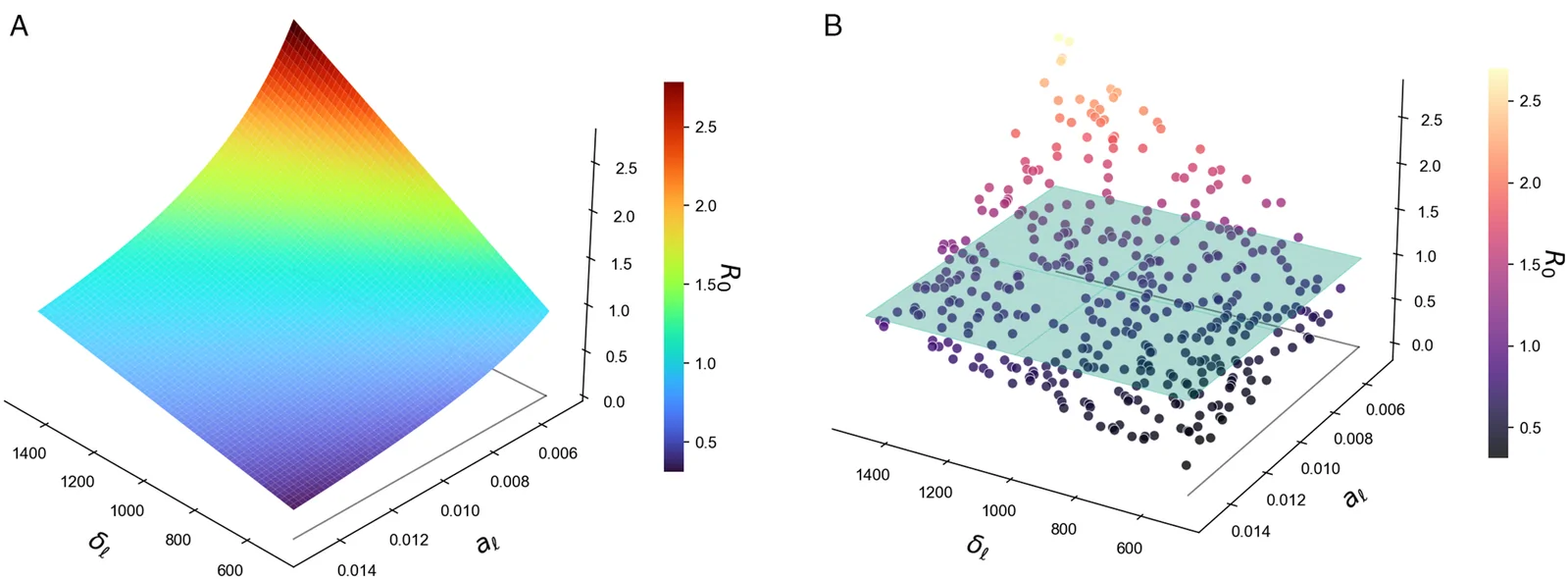
Threshold dynamics of the basic reproduction number ${ℛ}_{0}$ of system (1) under oncolytic virotherapy. The left panel illustrates the nonlinear dependence of ${ℛ}_{0}$ on viral burst size and clearance rate, while the right panel highlights the dispersion of ${ℛ}_{0}$ across the admissible parameter space, separating two distinct scenario for ${ℛ}_{0}<1$ and ${ℛ}_{0}>1$. (a) 3-D Surface plot of ${ℛ}_{0}$ as a function of the OV burst size $\delta_{l}$ and clearance rate $a_{l}$ (b) Scatter distribution of ${ℛ}_{0}$ with the *R*_0_ = 1 threshold plane highlighted.

Fig 6a presents a surface plot representation of the basic reproduction number ${ℛ}_{0}$ as a function of the oncolytic virus burst size $\delta_{l}$ and clearance rate $a_{l}$, which reveals a pronounced nonlinear dependence on therapeutic viral kinetics. An increase in viral production enhances ${ℛ}_{0}$, whereas rapid viral clearance suppresses it. Fig 6b in the right panel complements this observation through a scatter distribution which is generated using 1200 samples drawn uniformly from the specified interval ranges provided in Table 1, utilizing Monte-Carlo simulation method. It highlights the variability in ${ℛ}_{0}$ across sampled parameter combinations and illustrates transitions across the threshold plane. ${ℛ}_{0}>1$ here corresponds to enhanced proliferation of oncolytic virus-infected cancer cells, indicating effective therapeutic amplification, whereas ${ℛ}_{0}<1$ signifies viral fade-out and reduced treatment efficiency.

**Fig 7:**
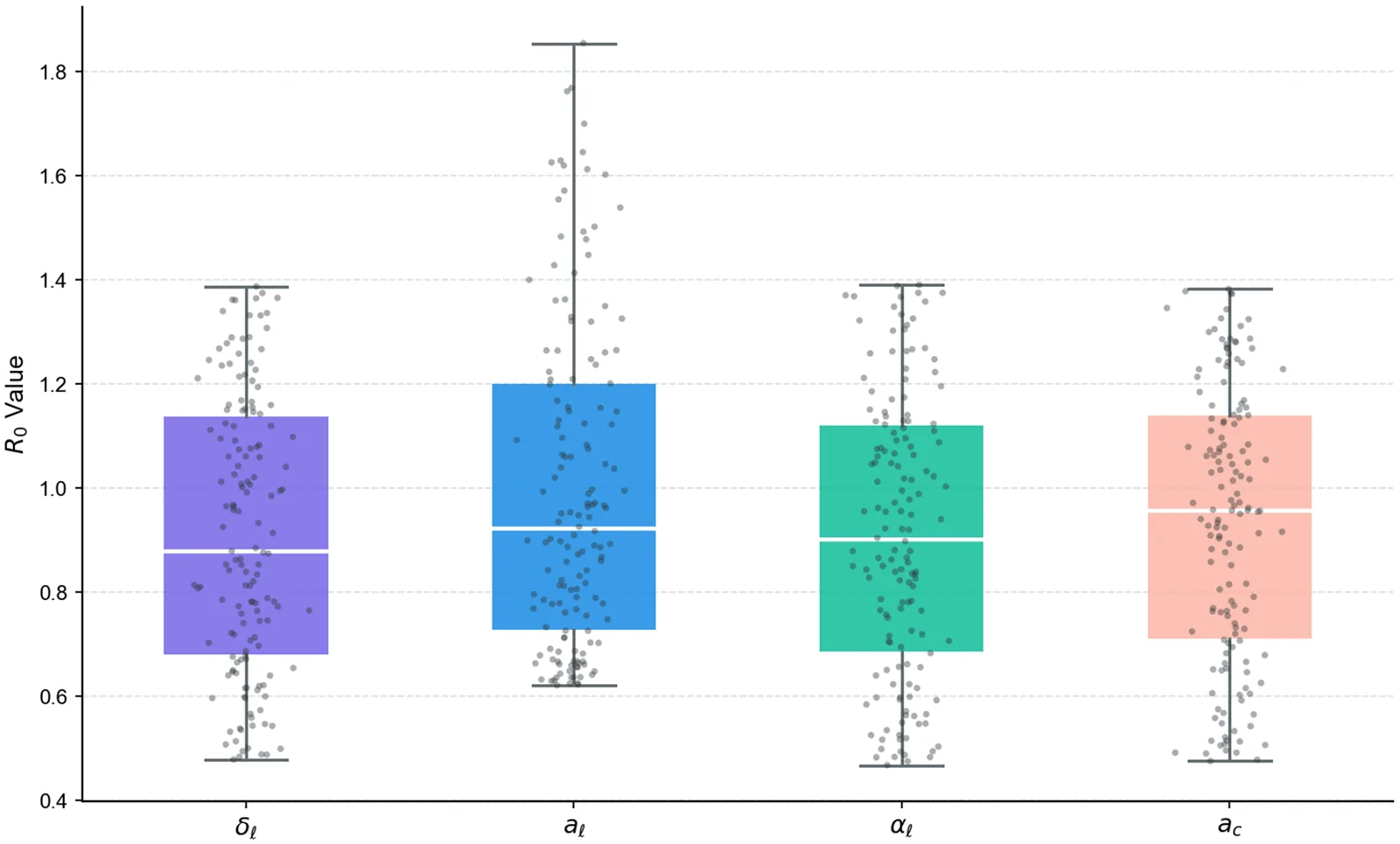
Box plot simulations exhibiting the distribution of the basic reproduction number ${ℛ}_{0}$ across parameter variations of $\delta_{l}$, $a_{l}$, $\alpha_{l}$, and $a_{c}$.

The box plots in Fig 7 depicts the variability in ${ℛ}_{0}$ values when the parameters $\delta_{l}$, $a_{l}$, $\alpha_{l}$, and $a_{c}$ are sampled across the specified biological ranges. The central white line within each box indicates the median ${ℛ}_{0}$, while the box boundaries show the inter-quartile range. It allows a direct assessment of how often ${ℛ}_{0}$ exceeds the critical threshold of 1. Whiskers indicate plausible extreme values of ${ℛ}_{0}$, i.e., how far ${ℛ}_{0}$ can spread beyond the core range without being considered as abnormal. Wider boxes and longer whiskers observed clearly for the parameters associated with virotherapy indicate enhanced variability in the reproduction threshold associated with viral amplification.

**Fig 8:**
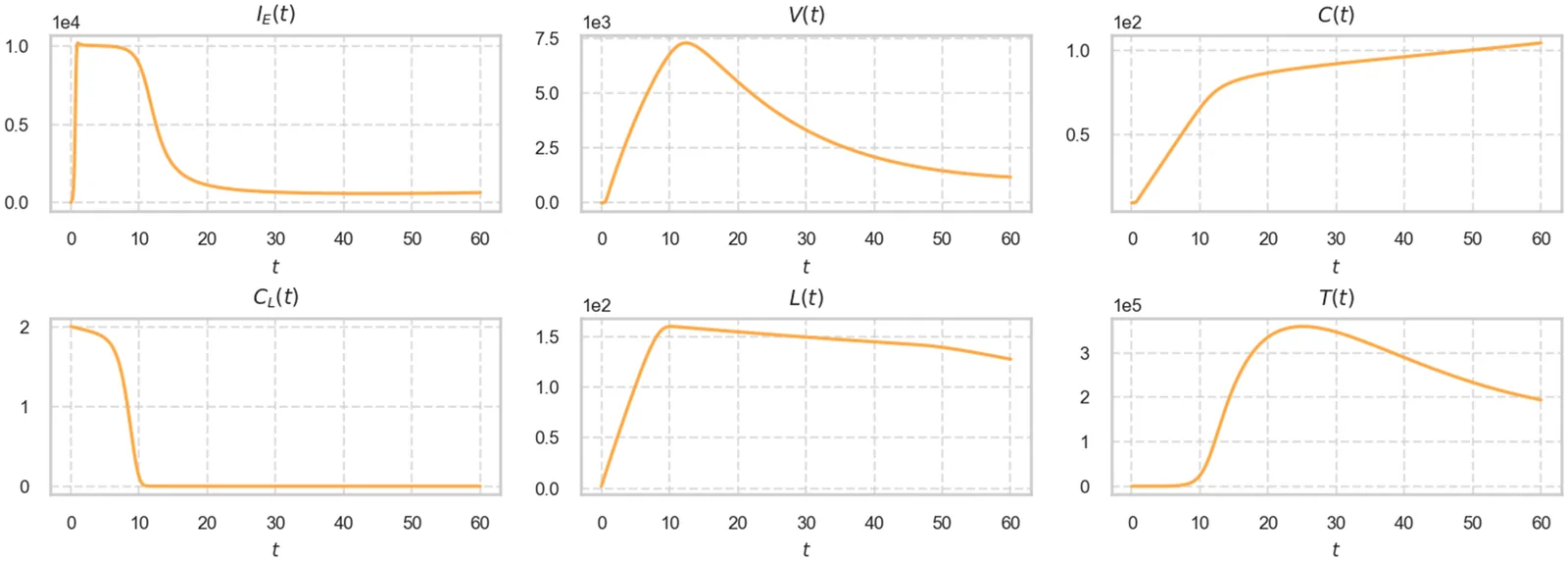
Optimal state dynamics of the controlled system (13) under the virotherapy-only control strategy (*S*_1_). This figure illustrates the temporal evolution of the state variables $I_{E}(t)$ (infected epithelial cells), *V*(*t*) (HPV virions), *C*(*t*) (cancer cells), $C_{L}(t)$ (OV-infected cancer cells), *L*(*t*) (oncolytic virus particles) and *T*(*t*) (cytotoxic T-lymphocytes). In this strategy, only virotherapy is applied, i.e., $u_{l}(t)\text{≠}0$ and $u_{m}(t)=0$, representing the absence of chemotherapy. The dynamics demonstrate the effect of virotherapy alone on tumor progression and viral activity within the host. The simulations are performed using the baseline parameter set provided in Table 1 where $\left[I_{E}(0),V(0),C(0),C_{L}(0),L(0),T(0)]=[1.0,\;1.0,\;30.0,\;2.0,\;2.0,\;5.0\right]$ are chosen as the initial conditions.

**Fig 9:**
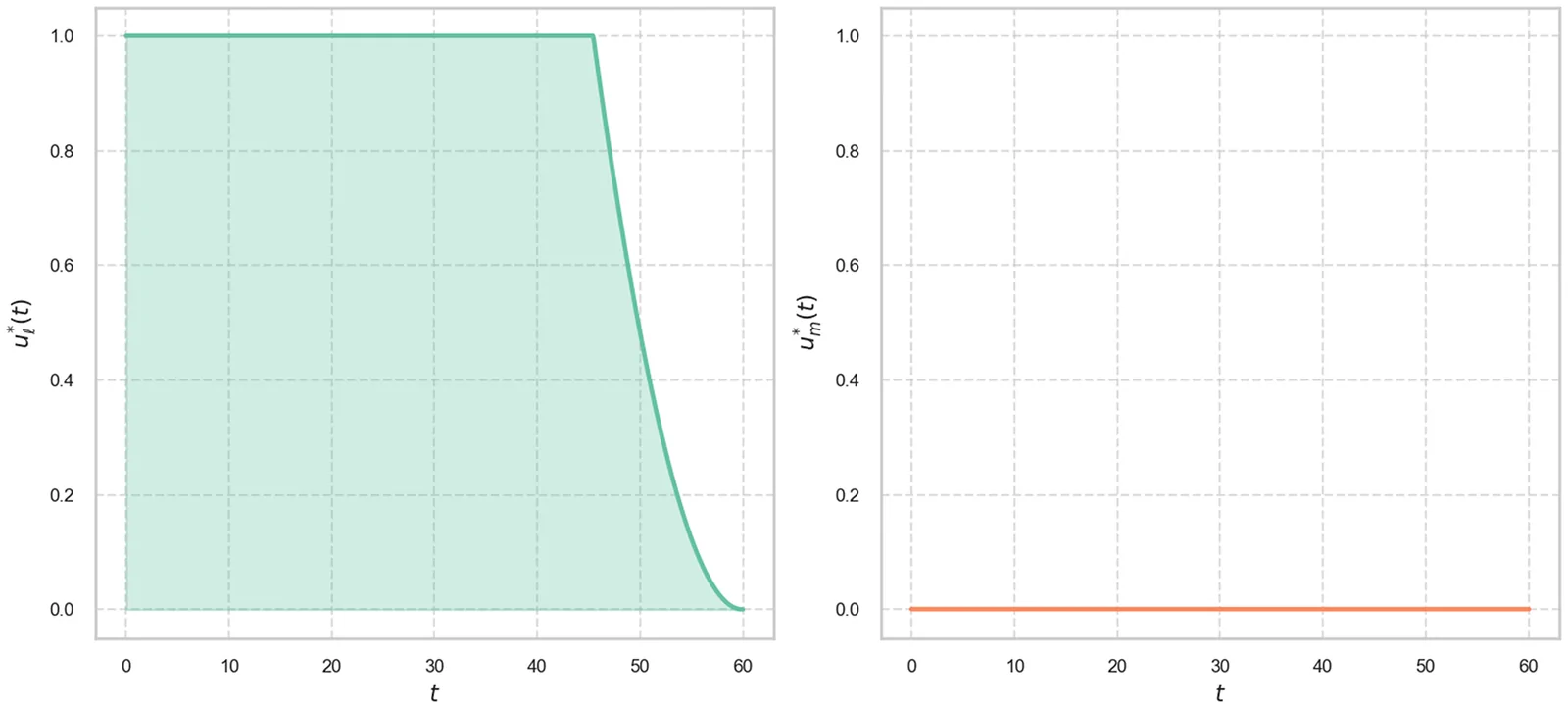
Optimal control profiles corresponding to the virotherapy-only strategy (*S*_1_) for system (13). The optimal virotherapy control $u_{l}^{*}(t)$ varies over time for a treatment period of approximately 60 days, while the chemotherapy control $u_{m}^{*}(t)$ remains identically zero throughout the treatment horizon.

Nextly, we have experimented with the nature of the trajectories of the control-induced system denoted as (13) under strategy *S*_1_, where the system is driven solely by virotherapy, with chemotherapy excluded from the treatment protocol. The resultant simulations are manifested in Fig 8 and the corresponding control profiles are demonstrated in Fig 9. The optimal control profile shows an initial phase of maximal virotherapy administration, followed by a gradual reduction after 45 days as the system approaches a controlled state, while the chemotherapeutic input remains inactive. This control structure leads to pronounced reductions in infected epithelial cells and virus-induced cancer cells, accompanied by sustained oncolytic virus activity and a delayed yet significant immune response as depicted in the subplots of Fig 8.

**Fig 10:**
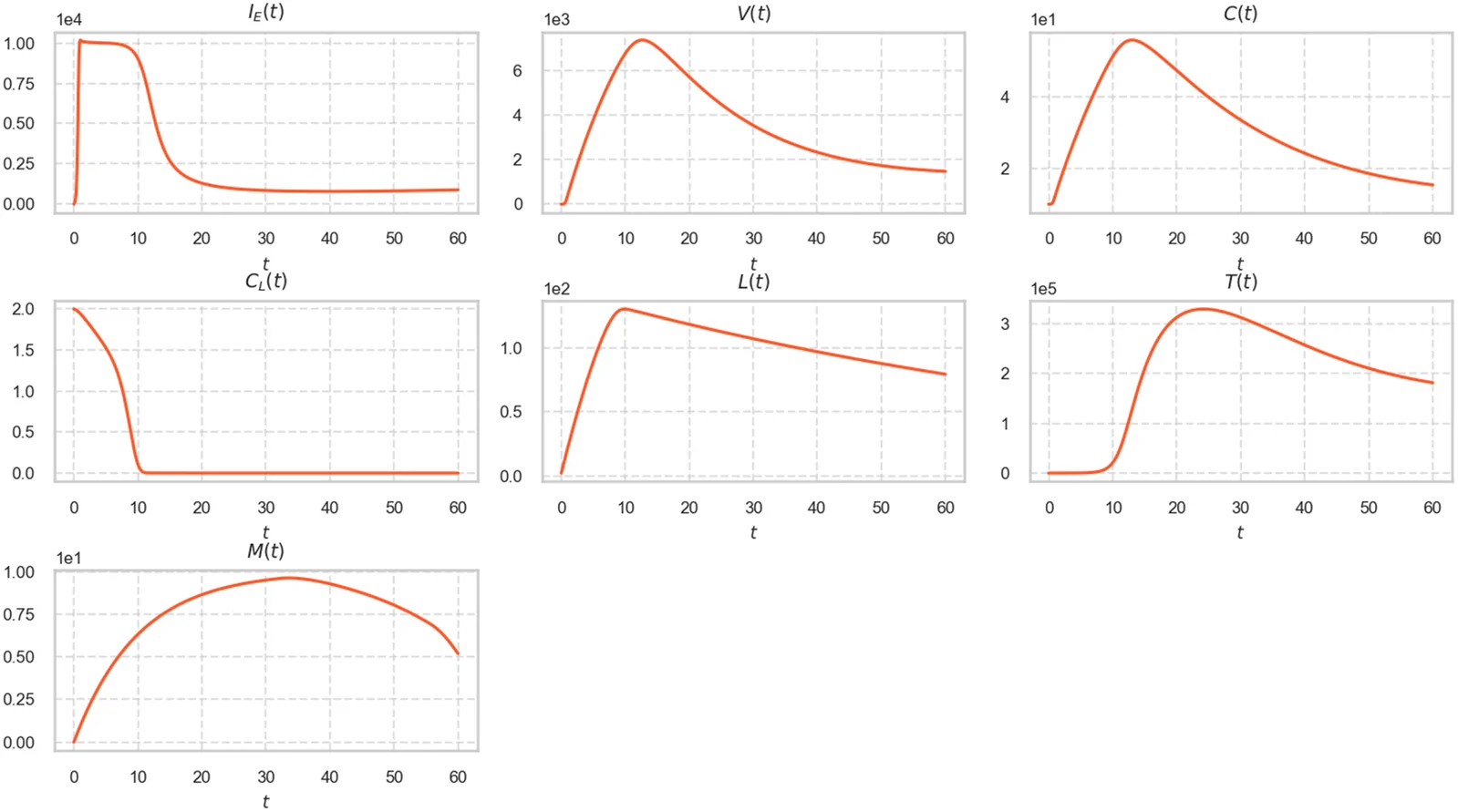
Time evolution of the system populations $I_{E}(t)$, *V*(*t*), *C*(*t*), $C_{L}(t)$, *L*(*t*), *T*(*t*), and chemotherapeutic drug concentration *M*(*t*) under the chemotherapy-only control strategy (*S*_2_) for system (13), where virotherapy is absent ($u_{l}(t)=0$). Here, $\left[I_{E}(0),V(0),C(0),C_{L}(0),L(0),T(0),M(0)]=[1.0,\;1.0,\;30.0,\;2.0,\;2.0,\;5.0,\;2.0\right]$ are chosen as the initial conditions for the simulation.

**Fig 11:**
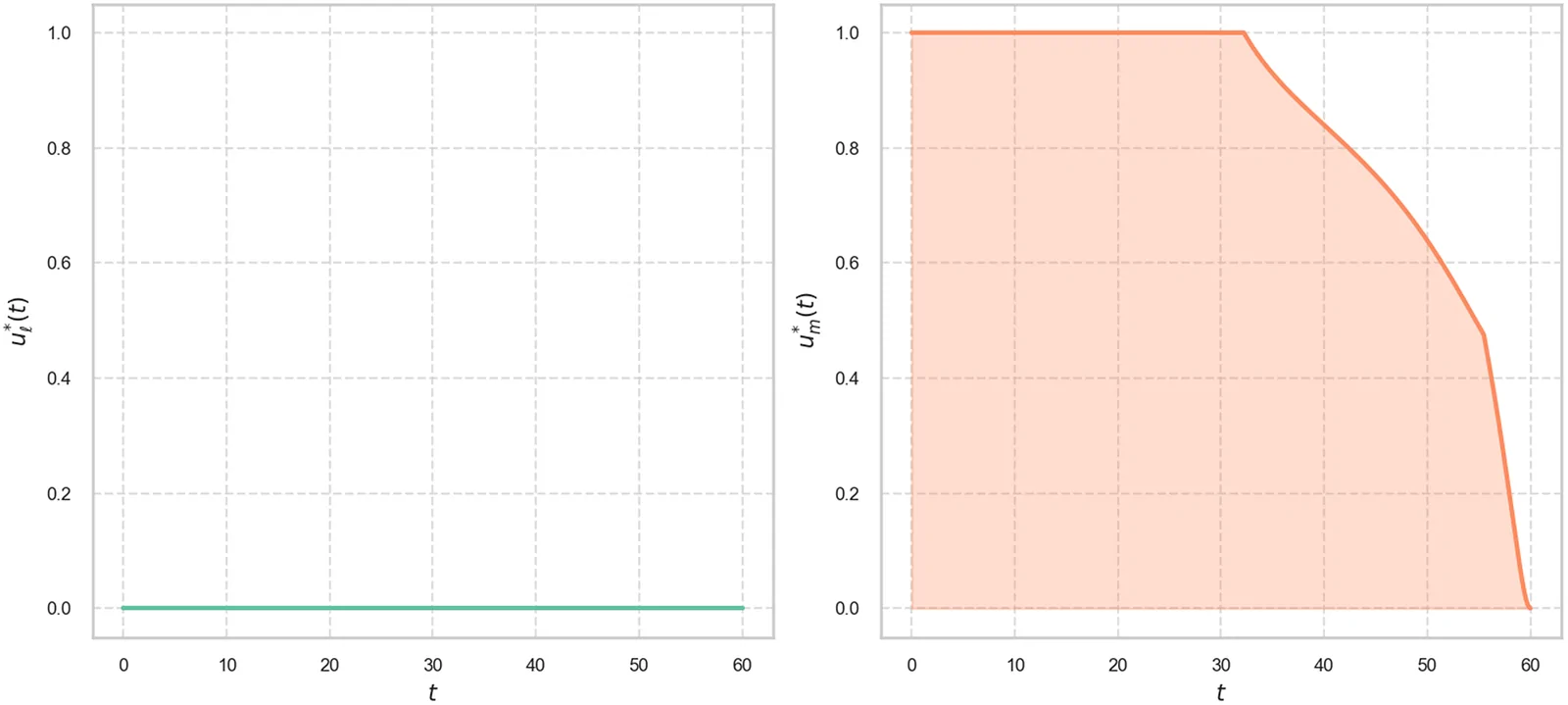
Optimal control profiles for system (13) corresponding to the chemotherapy-only strategy (*S*_2_), showing a time-varying chemotherapeutic input $u_{m}^{*}(t)$ while the virotherapy control $u_{l}^{*}(t)$ remains identically zero.

In contrast to the virotherapy-only protocol, strategy *S*_2_ explores the therapeutic consequences of administering chemotherapy as the sole intervention, with virotherapy administration set to zero. The controlled state trajectories for system (13) and the associated control profiles are shown, respectively, in Figs 10 and 11. The optimal control framework indicates sustained chemotherapy during the early phase, followed by a gradual tapering after 32 days as the treatment progresses. This dosing pattern drives a direct cytotoxic impact across multiple cell populations, reflected by reductions in infected epithelial and cancer cell levels, while the chemotherapeutic concentration exhibits a transient rise before declining due to drug decay. The absence of virotherapy administration, i.e., the input $u_{l}(t)=0$ leads to diminished viral and infected cancer cell dynamics, with immune activation arising primarily from residual disease burden.

**Fig 12:**
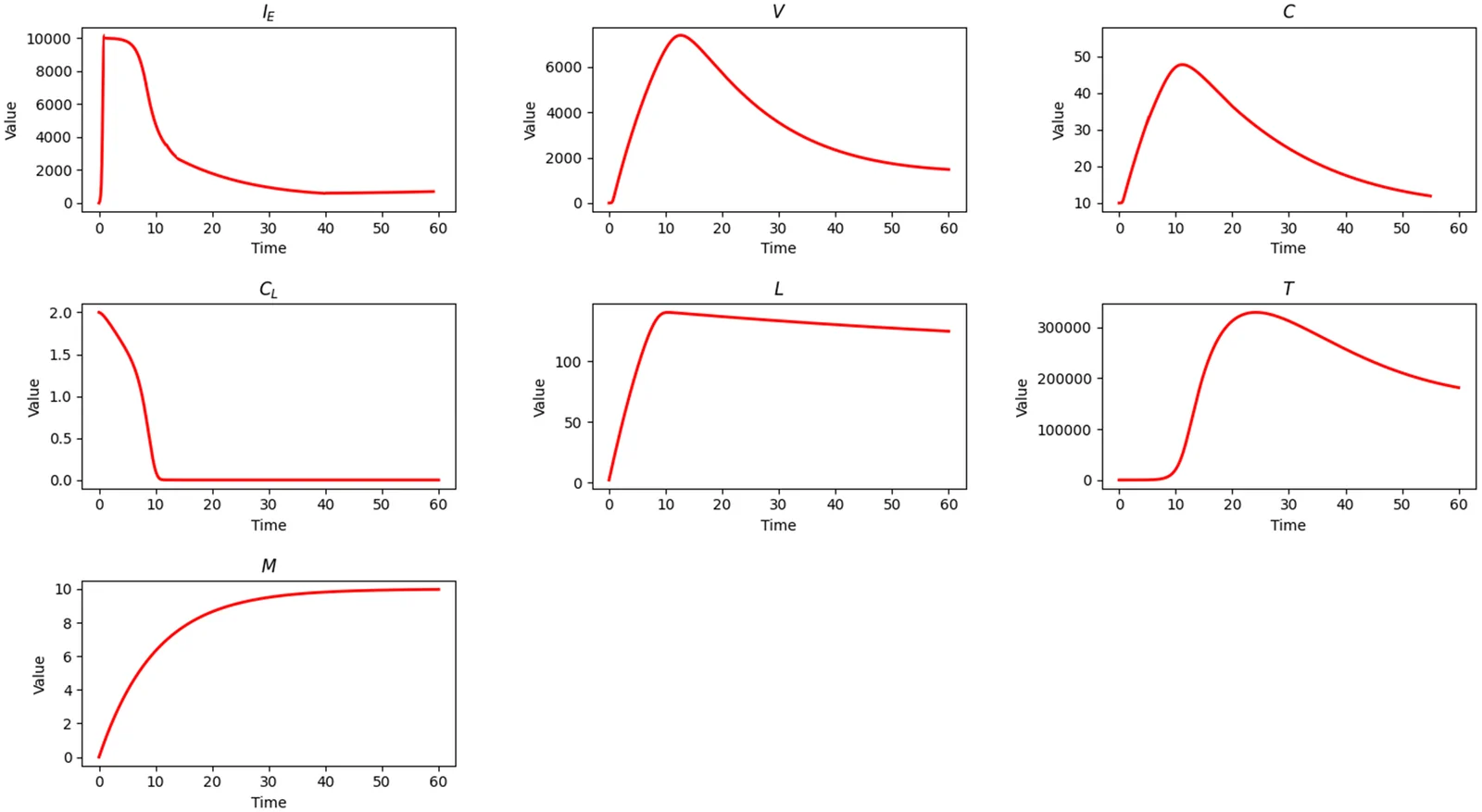
Time evolution of the state variables of the control-induced system (13) under the combined administration of oncolytic virotherapy and chemotherapy. The system trajectories illustrate the transient and long-term behavior of $I_{E}(t)$, *V*(*t*), *C*(*t*), $C_{L}(t)$, *L*(*t*), *T*(*t*) and *M*(*t*). The combined *t*herapeutic strategy significantly suppresses cervical cancer progression while regula*t*ing viral and immune dynamics over *t*he *t*reatment horizon of approximately 60 days. Here, the initial conditions are chosen as $\left[I_{E}(0),V(0),C(0),C_{L}(0),L(0),T(0),M(0)]=[1.0,\;1.0,\;30.0,\;2.0,\;2.0,\;5.0,\;2.0\right]$.

**Fig 13:**
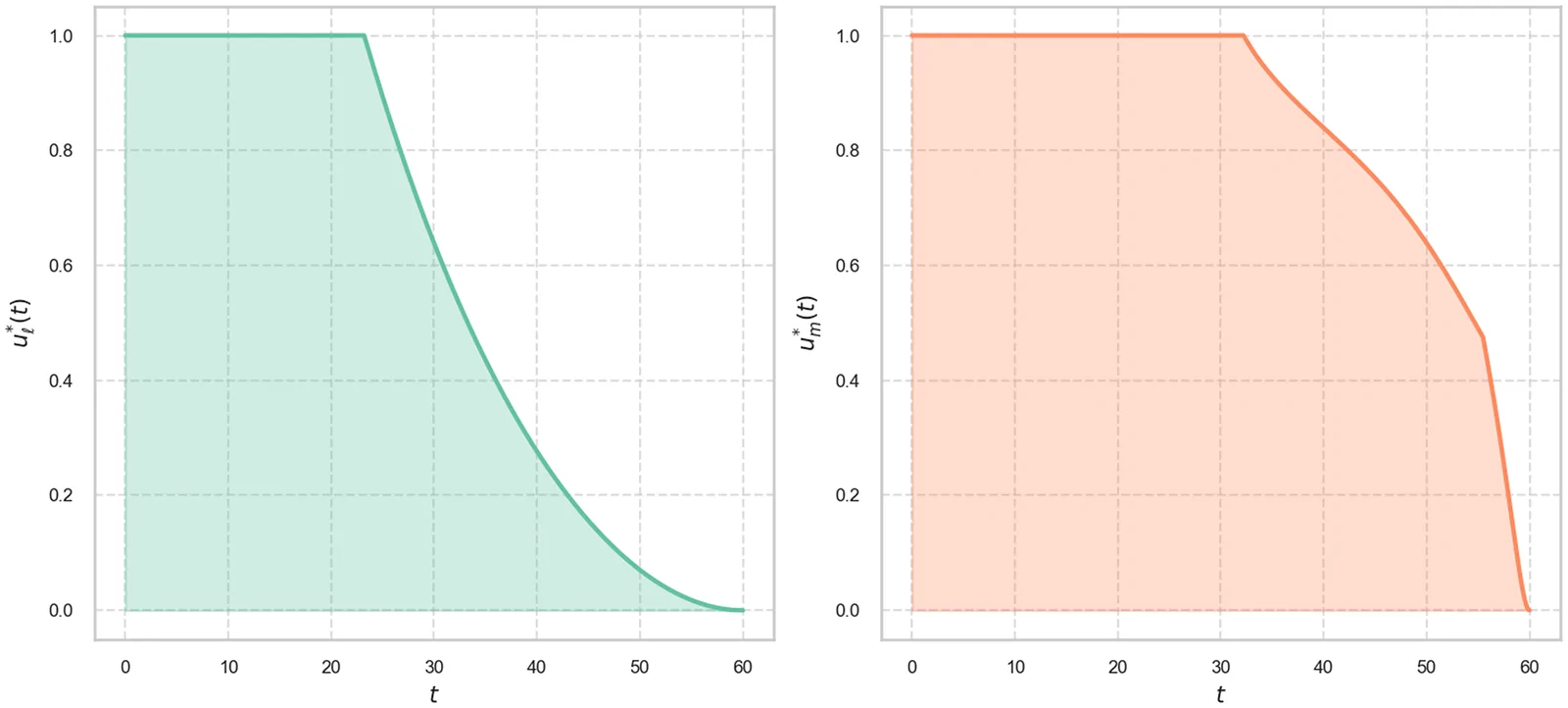
Optimal control profiles under the combined strategy *S*_3_ corresponding to the oncolytic virotherapy control $u_{l}^{*}(t)$ and the chemotherapeutic control $u_{m}^{*}(t)$ obtained from the optimal control problem defined for system 13. The profiles reflect an intensive early treatment phase and a reduced dosing strategy at later stages to balance the therapeutic efficacy as well as the treatment cost.

Simulations in Fig 12 indicate that the combined virotherapy-chemotherapy strategy, i.e., strategy *S*_3_ yields a marked reduction in cancer cell and infected epithelial cell density while maintaining regulated viral and immune responses. Both the infected epithelial cell population $I_{E}$ and the cancer cell density *C* initially rise due to disease progression but decline thereafter as therapeutic effects become dominant. A transient peak is observed in the viral load *V*, representing active viral replication, followed by a gradual decrease as susceptible target cells are exhausted. During treatment, the oncolytic virus density *L* increases and subsequently stabilizes at a lower level, whereas the CTL population *T* exhibits a pronounced reduction after reaching its maximum concentration. Meanwhile, the chemotherapeutic drug concentration *M* approaches a steady state under sustained administration. The optimal control profiles $u_{l}^{*}(t)$, $u_{m}^{*}(t)$ depicted in Fig 13 further show that both therapies are applied at their maximum admissible levels in the early phase to suppress the infection, after which control intensities decrease smoothly, reflecting an optimal trade-off between therapeutic efficacy and treatment cost.

**Fig 14:**
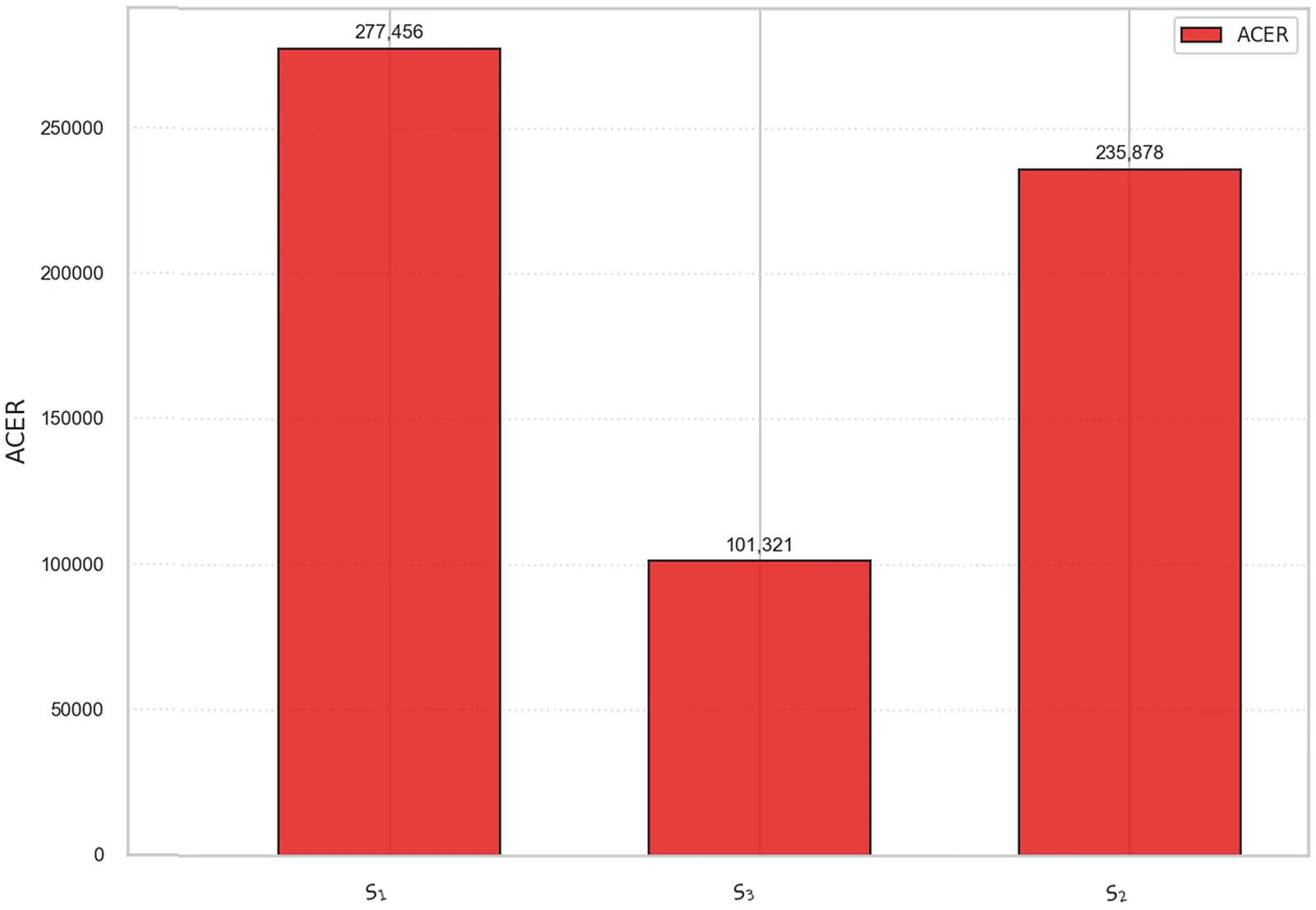
Average Cost-Effectiveness Ratio (ACER) for three control strategies: Virotherapy only (*S*_1_), Chemotherapy only (*S*_2_) and combined therapy (*S*_3_). Lower ACER values indicate more cost-effective strategies.

Fig 14 depicts the vertical bar plot of the Average Cost-Effectiveness Ratio (ACER) for the controlled system (13) under different treatment strategies $S_{i}$ for *i* = 1,2,3. The ACER corresponding to the *i*-th strategy is defined as


$$[ACER{]}_{i}=\frac{{𝒥}_{i}}{{𝔼}_{i}},$$

where ${𝒥}_{i}$ denotes the total treatment cost obtained from the objective functional defined in equation (15), and ${𝔼}_{i}$ represents the effectiveness of the strategy. In the present study, effectiveness is quantified in terms of Quality-Adjusted Life Years (QALYs). It is derived from the measurement of reduction in the concentration of the infected epithelial cell population $I_{E}(t)$ and the cancer cell populations *C*(*t*) under the influence of different control strategies implemented for the control-induced system (13). The values of ${𝔼}_{i}$ for *i* = 1,2,3 calculated and utilized for *t*his simulation are listed as 0.7341, 0.1080 and 0.050, respectively.

From Fig 14, it is evident that strategy *S*_3_, corresponding to the combined virotherapy and chemotherapy intervention, yields the lowest ACER value. This indicates that the combined therapy achieves the greatest health benefit per unit cost among all strategies. Biologically, this result suggests that the synergistic action of oncolytic viruses and chemotherapy enhances the control of epithelial and cancer cell infection more efficiently than the mono-therapies, thereby improving quality-adjusted survival at a reduced average economic burden.

## 7 Discussion and conclusion

In this present section, we have discussed all the analytical and numerical findings of the proposed mathematical frameworks and the biological implications in the context of HPV-induced cervical cancer under oncolytic virotherapy and chemotherapy have been interpreted. We have emphasized on elucidating the threshold behaviour, stability properties, parameter sensitivity for system (1), and optimal therapeutic strategies associated with system (13), with the aim of translating the model outcomes into insights relevant for treatment design and comparative effectiveness assessment.

Analytically, solutions of system (1) are proved to be nonnegative and uniformly bounded in the positively invariant domain 𝒟. Thus, the biological plausibility and well-posedness of the system is established. We have shown that the oncolytic virus-free equilibrium is governed by the threshold ${ℛ}_{0}$, obtained as a result of applying the next-generation matrix method. In biological terms, ${ℛ}_{0}$ summarizes whether the OV-induced infection invades the cancer cell population leading to potential therapeutic enhancement. Regarding this, the distinct scenarios of ${ℛ}_{0}<1$ and ${ℛ}_{0}>1$ are later investigated in the study numerically in the subplots of Fig 6. The endemic equilibrium analysis further indicates that long-term coexistence can occur and that stability depends on the derived Routh-Hurwitz conditions.

Insights from the global sensitivity analysis depicted in Figs 2 and 3 underscore the dominant role of oncolytic kinetic parameters in shaping treatment outcomes. A novel algorithm for performing the global sensitivity through utilizing Extended Fourier Amplitude Sensitivity Test (eFAST) method is presented in detail in Section 4 denoted as Algorithm 1. The eFAST indices along with PRCC analysis, and ranked total-order sensitivities consistently identify the viral burst size, clearance rate, and infection rate of cancer cells as highly impactful parameters, while parameters associated with CTL-immune activation primarily exert influence through nonlinear interaction effects. These findings collectively emphasize that uncertainty reduction and therapeutic optimization are most efficiently achieved by refining estimates related to viral replication and persistence rather than uniformly adjusting all biological parameters.

The optimal control formulation bridges analytical findings with clinically relevant treatment strategies by allowing time-dependent administration of virotherapy and chemotherapy. The resulting control profiles for the combined strategy reveal a pronounced early intervention phase followed by a gradual reduction in dosing intensity after 25 days for $u_{l}^{*}(t)$, and after 32 days for $u_{m}^{*}(t)$ which captures a realistic balance between effective suppression of infected epithelial and cancer cell populations and the minimization of associated treatment costs. Under the combined therapeutic strategy, coordinated early dosing accelerates reductions in *C* and $I_{E}$, while maintaining regulated viral and immune dynamics throughout the treatment horizon. Although the combined strategy is shown to be the best possible choice for treating cervical cancer, in some specific types of cases the virotherapy only strategy can be implemented securely and successfully. When the cancer cell burden is relatively low into the patient’s body, lesions are localized and the disease is detected early, virotherapy can be a good choice as OV replicate best when cancer cells are accessible and not massively heterogeneous. Severe toxicity risks associated with chemotherapy-only strategy persists as a long term point of concern. There are plenty of evidences where patients have renal dysfunction, poor bone marrow reserve etc. and patients can not cope up with chemotherapy for a longer period of time [52,53]. In these situations, virotherapy becomes relevant as it exhibits relatively lower systemic toxicity than chemotherapy and local immune stimulation rather than systemic cytotoxicity. Lastly, virotherapy-only strategy can be considered as a safe alternative where immune responsiveness to HPV-infected epithelial and OV-infected cancer cells is pronounced, as reflected by enhanced CTL activation in response to infected compartments.

Thus, comparing the strategies, our study suggests that *S*_1_ (virotherapy only) can be effective but is more sensitive to cancer cell decline. Strategy *S*_2_ (chemotherapy only) imposes broad cytotoxic pressure yet lacks viral amplification. The combined strategy *S*_3_ benefits from complementary mechanisms and attains the lowest ACER, which indicates the best average health gain per unit cost among the tested options.

It should be noted that in the present study, the parameter values utilized in simulations are obtained from different relevant published sources rather than estimated from a single experimental dataset; therefore, the numerical simulations should primarily be interpreted as qualitative illustrations of the model dynamics rather than as experimentally calibrated quantitative predictions. However, this research work collectively establishes a quantitative modeling framework for evaluating and optimizing combined virotherapy-chemotherapy regimens against HPV-induced cervical cancer. The analysis underscores the critical roles of sustained oncolytic activity and efficient OV-induced infection of cancer cells, supporting a treatment paradigm of early intensive combination therapy followed by a controlled taper. With further refinement through improved biological parameterization and clinically relevant outcome measures, this approach can provide a practical tool for planning personalized treatment protocols and guiding strategic choices under varying clinical constraints.
